# Exercise blood-drop metabolic profiling links metabolism with perceived exertion

**DOI:** 10.3389/fmolb.2022.1042231

**Published:** 2022-12-21

**Authors:** Tobias Opialla, Benjamin Gollasch, Peter H. J. L. Kuich, Lars Klug, Gabriele Rahn, Andreas Busjahn, Simone Spuler, Michael Boschmann, Jennifer A. Kirwan, Friedrich C. Luft, Stefan Kempa

**Affiliations:** ^1^ Department of Proteomics and Metabolomics Max-Delbrück-Center for Molecular Medicine Berlin, Berlin Institute for Medical Systems Biology, Berlin, Germany; ^2^ Muscle Research Unit, Experimental and Clinical Research Center, A Joint Collaboration Between Max-Delbr ück-Center and Charité Universitätsmedizin Berlin, Berlin, Germany; ^3^ Berlin Institute of Health Metabolomics Platform, Charite Universitätsmedizin Berlin, Berlin, Germany; ^4^ Experimental and Clinical Research Unit, Joint collaboration between Max-Delbr ück-Center and Charité Universitätsmedizin Berlin, Berlin, Germany; ^5^ HealthTwiSt GmbH, Berlin, Germany

**Keywords:** gas chromatography, blood drop sampling, relative perceived exertion, hypoxia, metabolomics

## Abstract

**Background:** Assessing detailed metabolism in exercising persons minute-to-minute has not been possible. We developed a “drop-of-blood” platform to fulfill that need. Our study aimed not only to demonstrate the utility of our methodology, but also to give insights into unknown mechanisms and new directions.

**Methods:** We developed a platform, based on gas chromatography and mass spectrometry, to assess metabolism from a blood-drop. We first observed a single volunteer who ran 13 km in 61 min. We particularly monitored relative perceived exertion (RPE). We observed that 2,3-bisphosphoglycerate peaked at RPE in this subject. We next expanded these findings to women and men volunteers who performed an RPE-based exercise protocol to RPE at *Fi* O _2_ 20.9% or *Fi* O _2_ 14.5% in random order.

**Results:** At 6 km, our subject reached his maximum relative perceived exertion (RPE); however, he continued running, felt better, and finished his run. Lactate levels had stably increased by 2 km, ketoacids increased gradually until the run’s end, while the hypoxia marker, 2,3 bisphosphoglycerate, peaked at maximum relative perceived exertion. In our normal volunteers, the changes in lactate, pyruvate, *ß* hydroxybutyrate and *a* hydroxybutyrate were not identical, but similar to our model proband runner.

**Conclusion:** Glucose availability was not the limiting factor, as glucose availability increased towards exercise end in highly exerted subjects. Instead, the tricarboxylic acid→oxphos pathway, lactate clearance, and thus and the oxidative capacity appeared to be the defining elements in confronting maximal exertion. These ideas must be tested further in more definitive studies. Our preliminary work suggests that our single-drop methodology could be of great utility in studying exercise physiology.

## 1 Introduction

Physical exercise is healthy. (Castillo-Garzón et al., 2006; Warburton et al., 2006) The benefits are the same whether the exercise is recreational or occupational. (Lear et al., 2017) There are numerous assessment signs, including maximal oxygen consumption, muscle strength, muscular endurance, responses in heart rate, and others. (Nindl et al., 2015) Exercisers experience regular cycles of physiological stress accompanied by transient inflammation, oxidative stress, and immune perturbations. (Herrmann et al., 2015; Loprinzi, 2015; Palacios et al., 2015) The relevance of such findings to normal healthy individuals is not always clear. Furthermore, combining these diverse variables into an understandable paradigm is difficult.

Metabolomics is the scientific study of chemical processes involving metabolites, including an assessment of the unique chemical fingerprints that specific cellular processes leave behind, following a metabolic event. (Bassini & Cameron, 2014; Gong et al., 2017) The metabolome represents the collection of all metabolites in a cell, tissue, organ or organism, which are the end products of cellular processes. Gas chromatography–mass spectrometry (GC–MS) based metabolomics, as well as other technologies, now enable us to vastly increase our panoramic inspection of these processes. (Gong et al., 2017)

The field of exercise metabolomics is at its beginning. Klein and others reviewed a number metabolomics studies of bio-fluids and describe analytical platforms (Klein et al., 2021). The most comprehensive analysis of molecular changes post exercise was published recently by Contrepois and others (Contrepois et al., 2020). In a number of individuals metabolites, proteins and mRNA expression was studied post-exercise and all measured parameters were correlated to insulin resistance or sensitivity. Studies so far usually describe metabolic changes post exercise in plasma or serum.

Concurrently, we were interested in developing an analytical strategy that allows a simplified (dropwise) blood sampling that can be generally applied clinically or even to monitor subjects at or in the field. We have used a liquid–liquid sampling method for whole blood sampling that stabilizes the metabolome instantly and is optimal to monitor a broad spectrum of intermediates from central metabolism that represents the energy providing machinery. Our methods brings metabolomics into the practicable clinical arena.

Applicability commonly results after initial clinical observations. We performed initial observations in a model proband runner (MPR) who recorded his relative perceived (admittedly subjective) exertion (RPE). (Borg, 1982) Non-etheless, we had obtained an “*n*-of-one” dataset. We therefore aimed to investigate the practical utility of metabolomics. We extracted nine key metabolic features that seem to describe the feeling states observed in RPE on a personalized level. Many of these nine metabolic features are directly involved with oxygenation status. To test our observation that the subjective state, RPE, is reflected in the metabolome in a wider population, we designed a follow-up study in normal, recreationally active, women and men across a broad fitness and age spectrum, to inspect metabolomics outcomes.

We chose alterations of oxygen availability as additional stressor corresponding to normobaric-altitude sea level *versus* simulated 3,000 m (*Fi* O _2_ 14.5%) in a randomized setting, which is also employed in terms of fitness strategies. (Hobbins et al., 2017) We found that RPE is reflected in the metabolome in a wider population and underscored the ratios for liver metabolism previously established in 1967 by Krebs and associates. (Krebs, 1967; Williamson et al., 1967)

## 2 Results

**FIGURE 1:**
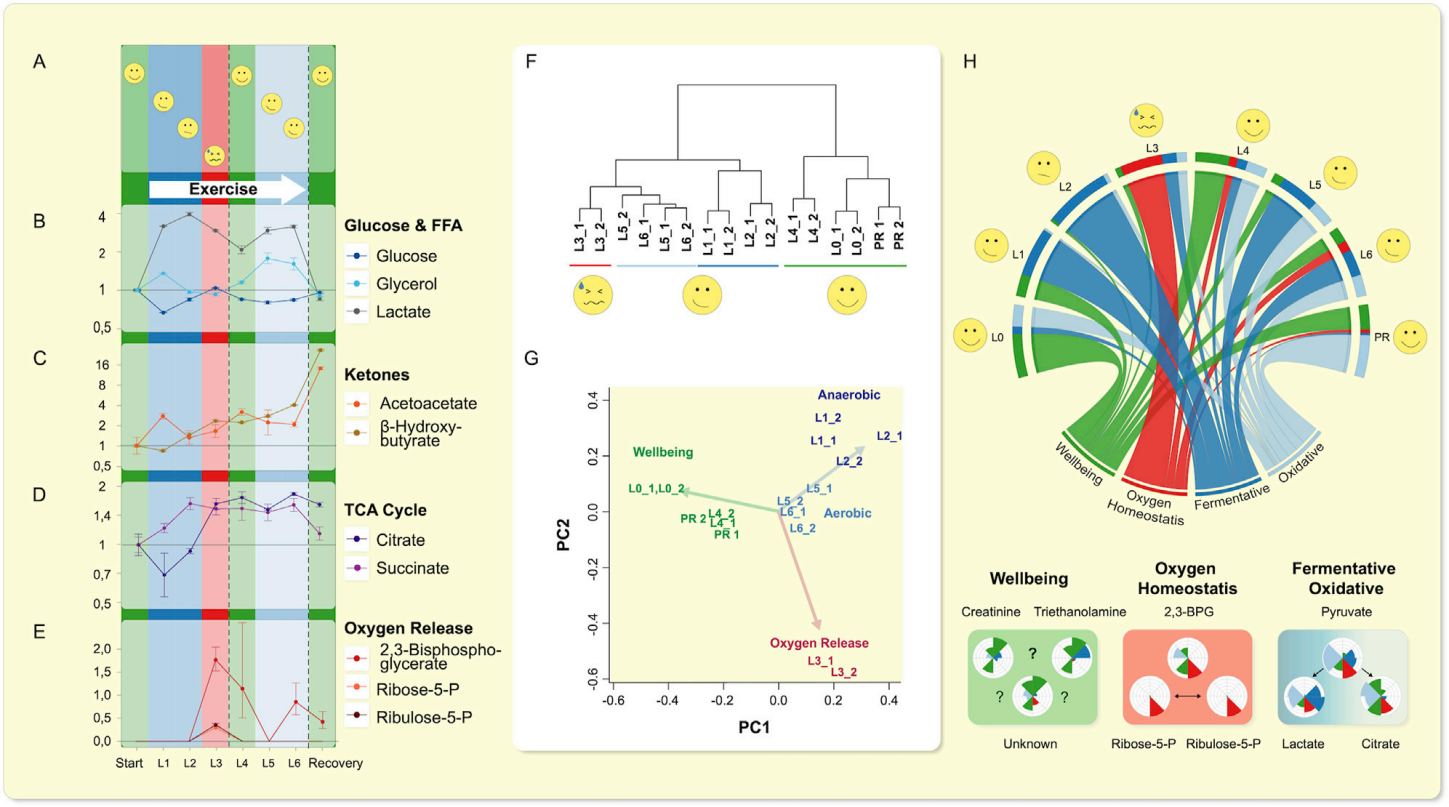
Single drop-blood analysis from an exerciser. **(A–E)** The rating of perceived exercise (RPE) variable is displayed as a subjective scale. Lactate (as opposed to glucose) increases similar to TCA-intermediates (Supplemental Figure S14), except for citrate. **(D)** Citrate only increased when oxygen release was promoted as indicated by rise of 2,3-BPG and PPP-intermediates Ribulose-5P and Ribose-5P above LOQ. **(E)** Glycerol showed two phases (corroborated by fatty acids, Supplementary Figure S15) of release and reaches baseline value, while **(B)** ketone bodies accumulate. **(F)** Hierarchical clustering of the technical replicates throughout the run on metabolic features is shown. Clustering, using all polar metabolites, grouped the sampling time-points in accordance with the volunteer’s self-perception. Lap 3 (L3), in which exhaustion occurred, was least related to beginning recovery (PR), and the most at maximum RPE (L4, Lap 4), while these three timely very diverse laps were closest related. **(G)** Visualization of a nine-metabolite principle-component analysis found by factor analysis representing the explanatory components of all data (relative activity of anaerobic and TCA cycle activity (pyruvate/lactate vs. pyruvate/citrate), oxygen release, and oxidative stress, as well as markers of “feeling energetic” are shown. These metabolites reproduced the principal relationships of metabolic states and transitions as observed within all data. **(H)** The visualization of the progression through the exercise regime is summarized in a *circos* plot (Krzywinski et al., 2009) using the nine explanatory metabolic features and relationships depicted in Nightingale plots below. The *circos* plot shows progression of each of the four factors determined in **(G)**, both for each factor (lower “root”) and through each lap (start at left “L0”). The Nightingale plots below show the progression of the nine key metabolites (see main text). Beginning at rest, the subject entered an initial anaerobic phase, which was followed by an energy crisis caused by insufficient oxygen availability. Resolution in Lap 4 was accompanied by a transient increase in indicators of feeling “energetic” (i.e. low RPE) and immediately followed the successful transition to oxidative TCA cycle driven energy supply that remained elevated until after exercise completion.

Our MPR (Figure 1 and Table S1) ran six laps (about 13 km in total) over an irregular terrain. He reported his perceptions of energy availability on a qualitative exertion scale (relative perceived exertion) RPE scale (Figures 1A–E). (Borg, 1982) Our subject was confronted with exhaustion on lap 3. He recovered, and remained approximately at the same performance level till the end of the run. In the drops of blood, we annotated 276 separate peaks of which 93 were identified using a comparison mix of commercial standards analyzed in the same batch (Table S2) (see methods and Opialla et al., 2020) giving a general central carbon metabolism coverage (Supplementary Figure S1). Lactate levels increased early in our subject, glucose remained flat, acetoacetate and *ß*-hydroxybutyrate increased progressively, while 2,3-biphosphoglycerate (2,3-BPG) peaked in lap 3 (Figures 1B–E). Succinate and other TCA intermediates also rose early, except for citrate, which only increased concurrently with 2,3-BPG levels. When we performed unsupervised hierarchical clustering, we were surprised that the samples clustered according to feeling state (“I am done”/“I feel ok”): most related to baseline and recovery; namely relative perceived exertion (RPE, Figure 1F), and not as one might expect according to exercise status.

We explored the co-behavior of metabolic changes and subjective feeling states of our MPR. Using a factor analysis we identified nine key metabolites that possessed the highest eigen-values. Based on these nine key metabolites, we were able to reproduce the clustering (Figure 1G, Supplementary Table S1) of our subject’s “feeling” states. We assigned physiological states to the metabolic features and set the dynamic changes in relationship to each other and show them in a *circos* plot (Krzywinski et al., 2009) (Figure 1H). Beginning at rest, the subject entered an initial anaerobic phase, which was followed by an episode likely caused by insufficient oxygen availability. The resolution was accompanied by a transient increase in indicators of feeling more robust and immediately followed the successful transition to oxidative Krebs’ tricarboxylic acid cycle (TCA)-driven energy supply, that remained elevated until after exercise completion (Figure 1H). We observed that creatine and triethanolamine were elevated when perceived maximum effort was “overcome”. We interpreted the 2,3-bisphosphoglycerate, ribose-5-phosphate, and ribulose-5-phosphate levels as indicating changes in oxygen homeostasis, and pyruvate, lactate, citrate ratios, as a switch between anaerobic and aerobic metabolism. Since 2,3-bisphosphglycerate is involved in shifting the oxygen-hemoglobin-saturation curve rightward (Benesch & Benesch, 1967; Chiba & Sasaki, 1978), we connected the results into our subsequent research plan.

**FIGURE 2:**
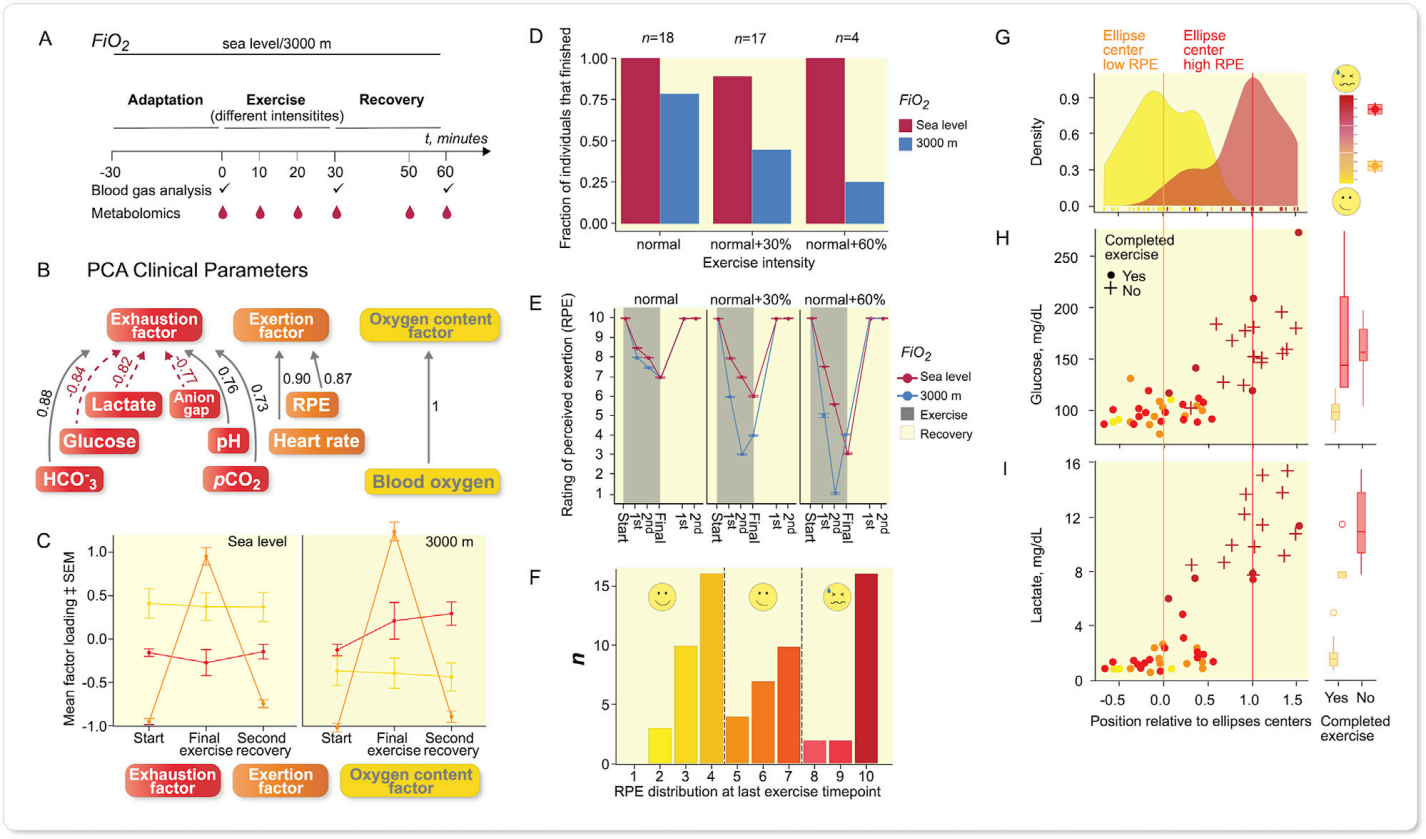
Results from recreational (normal) volunteers. Sampling scheme for larger panel study of recreational athletes. **(A)** Study design outline. **(B)** PCA of clinical parameters revealed three different items, namely exhaustion, exertion, and oxygen. **(C)** When plotting mean factor loadings of PCA of the three principle components, the data indicate that the oxygen content factor was influenced by *Fi* O _2_ albeit constant throughout the experiment. Exertion was high at last exercise time-point and reaches baseline values during recovery, while the exhaustion increases under more hypoxic conditions and remains elevated during recovery, error bars: SEM, *n* = 39 at each oxygenation level. **(D)** The fraction of subjects being able to complete the exercise bouts reflects that *Fi* O _2_ had a stronger influence on that with increasing power output (running speed), *n* given in the figure refers to each oxygenation level. **(E)** Individual RPE values were more different between power output levels; lower *Fi* O _2_ led to more marked exertion according to rating of perceived exertion (RPE), error bars: SEM, n-numbers as in **(D)**. **(F)** Histogram of RPE-distribution at last exercise time-point revealed a trimodal distribution, which was not explained by power output or *Fi* O _2_ alone (see Supplementary Figure S4). **(G)** PCA of metabolite level-fold changes final exercise time-point vs. baseline from experiments leading to distinct levels of RPE, revealed a separation of less exerted individuals from highly exerted individuals; density plot shows distribution along vector between confidence ellipses’ centers (Supplementary Figure S5). Glucose **(H)** and lactate **(I)** were major contributors to the separation. A + indicates that exercise was not completed, • shows exercise completed, color reflects RPE at last exercise time-point. **(G–I)**: low RPE: *n* = 29, high RPE: *n* = 20.

To study metabolism at exercise further (Figure 2) and the influence of oxygen availability, we next recruited 26 normal women and men volunteers across a broad age and fitness-level spectrum (Supplementary Table S3), who were randomized (cross-over) to perform at oxygen levels (*Fi* O _2_) at sea level or at a simulated 3,000 m altitude and at a running speed corresponding to 65% of maximum power output according to Jones *et al*. (Jones et al., 1985) (Figure 2A). The subjects all lived in the area of Berlin, Germany (≈50 m NN), reported various degrees of compliance to accepted healthy life styles and a broad gamut of physical fitness activities ranging from very little training to dedicated daily fitness schedules. The “normal” running speed often was not appropriately challenging to some subjects. As we were interested in a state of high RPE (“exhaustion”), we encouraged repetition of the experiment on another day with increased running speed at 30% or even 60% faster (Supplementary Table S4).

The individual and mean exertional-related effects (Supplementary Table S5) show a substantial load on most subjects and demonstrated, that a 30% increase in effort and/or hypoxia were successful challenges (Figures 2D,E). Principal component analysis (PCA) shows the clinical variables in relation to exhaustion, exertion, and oxygen partial pressure (Figure 2B). A Spearman ranking of the variables is given (Figure S2), and a comparison between sea level and 3,000 m (Figure 2C). The exhaustion factor was certainly influenced by altitude as was the oxygen factor. The change in individual variables with exercise at two performance levels and two altitudes are most visible in the arterial blood gases, pCO_2_, anion gap (AG), and lactate values (Supplementary Table S5, Supplementary Figure S18). The fraction of persons completing the run decreased at the different exercise levels (Figure 2D), indicating that our normal recruits also commonly experienced their limits (Figure 2E). Obviously, there was a relationship between rating of perceived exertion (RPE), running speed, and oxygenation. Since the formula according to Jones *et al.* (Jones et al., 1985) does not accommodate for the subjects’ training status, we looked deeper into RPE and found that differences in *Fi* O _2_ only made a substantial difference at 30% running speed and a higher level (Figure 2E).

We found a trimodal distribution in RPE at final exercise time-point based on 77 runs (Figure 2F). These results were not based on performance level or *Fi* O _2_ alone (Supplementary Figures S3,4). Since RPE is somewhat subjective and because we wanted to investigate reflection of feeling state (RPE) in the blood metabolome, we concentrated on the runs finishing at low RPE and high RPE in the further analysis (55 runs total). After removing strong outliers according to Hotelling’s-*T* ^2^-test we were left with 46 samples. Grouping of the data revealed a separation of subjects with highest RPE-values from those with lowest RPE-values (Figure 2G, Supplementary Figure S5), indicated by very little overlap of 95% confidence ellipses. Glucose and lactate values showed corresponding increases (Figures 2H,I). These unsupervised analyses were performed on fold changes between baseline and final exercise time-point in the metabolomics data set (122 identified peak species across all samples). We extracted the contributions of individual metabolites along the vector between the 95% confidence ellipse’s centers, similar to a dogleg plot of principal components. We could not underscore a role of 2,3-bisphosphoglycerate, but interpreted these data as indicating that glucose availability was not among rate-limiting factors.

**FIGURE 3:**
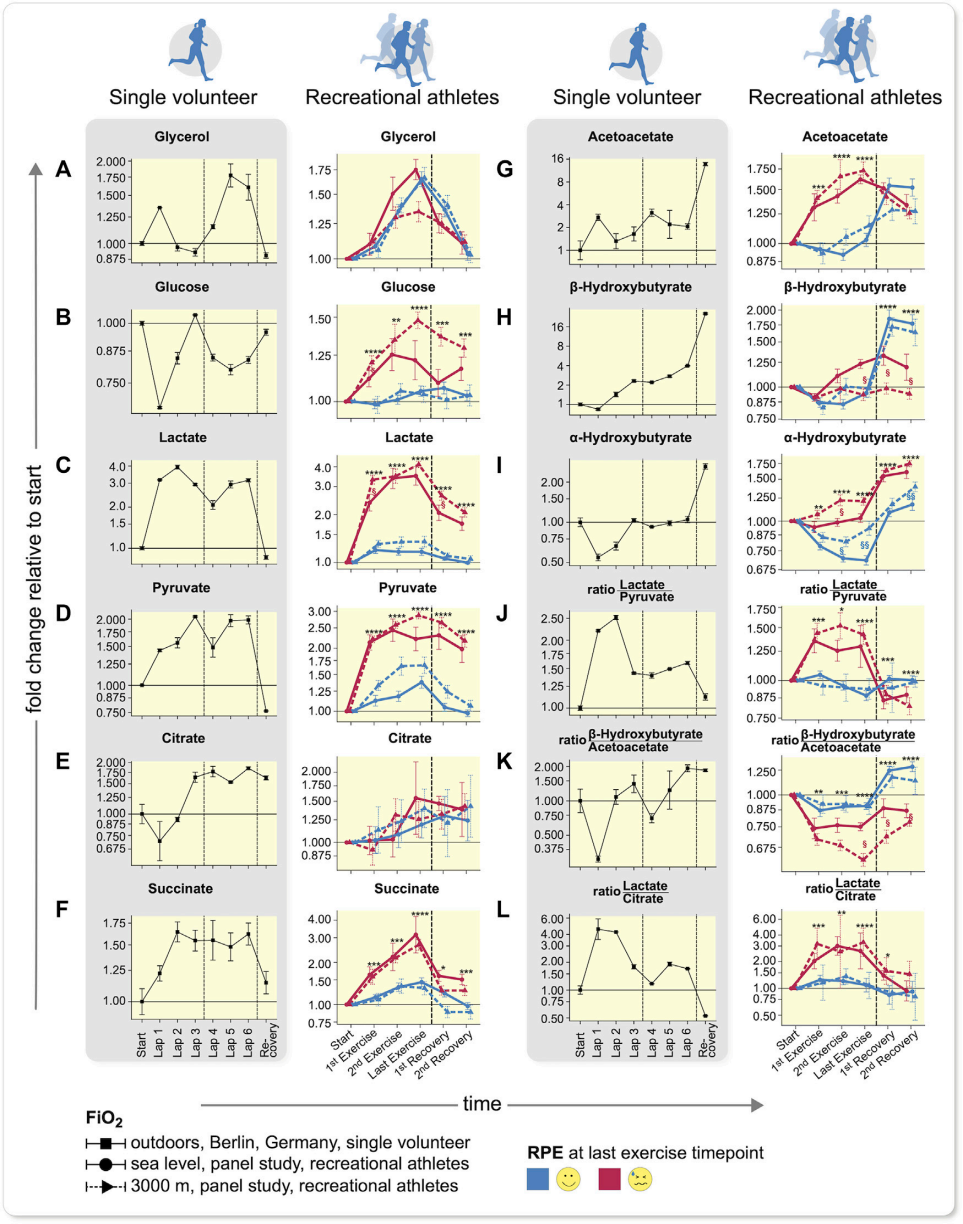
Time-profiles throughout exercise to recovery comparing single volunteer to recreational athletes. **(A–F)** Carbohydrate-based metabolite values, **(G–I)** ketone bodies, and **(J–L)** metabolite ratios in a single volunteer (left, error bars demonstrate deviations of duplicate measurments to anticipate the measurement accuracy) and recreational athletes (right) are shown (mean ± SEM). Difference between two technical replicate measurements are grouped according to *Fi* O _2_ (solid: sea level, dotted: 3,000 m) and RPE at last exercise time-point. Dashed lines encompass comparable time intervals in different setups. In panels on the left only one recovery time-point was measured, while in the RPE right panels exertion was not overcome; however, exercise was discontinued after 30 min. The samples between the vertical lines in the shaded, left plots depict a state, after overcoming subjective exhaustion that could not be compared to samples from the larger cohort study (*p*-values from two sided Wilcoxon-rank-sum (Benjamini-Hochberg FDR-corrected), *p < 0.05; **p < 0.01; ***p < 0.001; ***p < 0.0001 of all values within one RPE-group, for clarity only significance vs. lowest RPE group is shown, but all comparisons were accounted for *p*-value correction, ^§^ *p* < 0.05; ^§§^ *p* < 0.01 between hypoxia and normoxia of lowest RPE group. Explanations see main text. Low RPE: *n* = 29, high RPE: *n* = 20).

We next more closely examined the main contributing metabolites and plotted their time-profiles of intensity-fold changes relative to exercise start from start to recovery (Figure 3). The data of our single runner and recreational volunteers are clearly delineated. We were interested in dissecting the mechanism involved in self-perceived maximal exercise. Since we observed a separation in the PCA according to RPE (Figure 2G, Supplementary Figure S5) and to a lesser extent to *Fi* O _2_ (Figure S7B), we separated the time-profiles (Figure 3) according to RPE-group (red/blue) and *Fi* O _2_ (solid/dashed lines). Significance values are indicated by asterisks (*) and significance represent FDR-adjusted (Benjamini–Hochberg) *p*-values from Wilcoxon-tests between RPE-groups, § denotes false-discovery rate (FDR)-adjusted *p*-values from Wilcoxon-tests between *Fi* O _2_ levels within the respective RPE-groups. The separation observed in PCA (Supplementary Figures S5,6) was mostly caused by glucose (Figure 3B), lactate (Figure 3C), and pyruvate (Figure 3D), while citrate values (Figure 3E) appeared less so. The ketone bodies, acetoacetate (Figure 3G) and *a*-hydroxybutyrate (Figure 3I), other sugars and polyols such as mannose, fructose, threonate, sorbitol, as well as alanine (Supplementary Figure S19), tri-ethanolamine-phosphate, TCA-intermediates, such as succinate (Figure 3F and Supplementary Figure S6) and glucose-6-phosphate (Supplementary Figure S20) also appeared discriminatory. Some separation according to *Fi* O _2_ was observed (Supplementary Figure S7B); however, exercise bouts under hypoxia, in which where RPE was low, group well with normoxia samples where RPE in general was less challenged. We interpret this result as indicating that exercise under hypoxia tends to lead to higher RPE. Other factors such as sex (Supplementary Figure S7C) or training state (Supplementary Figure S7D) showed no separation. However, when examining the main separator in our recreational volunteers, namely glucose, the data showed a strong separation between *Fi* O _2_ levels in individuals with high RPE.

Some individuals with higher RPE were not clearly separated from the less exhausted group. We filtered out those in highest RPE and already separated, and performed PCA again while keeping all RPE groups (Supplementary Figures S8–S10). Those individuals in the highest RPE, but not separated entirely from the lower exertion groups, were now separated from the subjects in the lower exertion groups along PC1 (Supplementary Figure S11). These remaining samples in the high RPE-group exhibited a similar but less pronounced phenotype for glucose and lactate (Supplementary Figure S11).

In accordance with our data with increasing exercise intensity, the amount of fat oxidized remained constant, while the additional energy is derived from glycogen and glucose. (van Loon et al., 2001) The glycerol values in our studies serve as a marker for fatty acid mobilization and were similar across all groups identified by RPE at the last exercise time-point (Figure 3A and Supplementary Figure S13). In hypoxia under high RPE less glycerol was mobilized, indicating a lower ability to oxidize fatty acids. In our athlete (MPR) (Figures 3A–C), an initial increase in glycerol and slight decrease in glucose was observed; however, when he reached his maximum RPE (Lap 3), these values had returned to baseline. In a second phase, while the TCA cycle was running (Supplementary Figure S14), glycerol again increased together with an increase in fatty acids (Supplementary Figure S15) and fatty acid-derived ketone bodies acetoacetate (Figure 3G), as well as *ß*-hydroxybutyrate (Figure 3H). His lactate level quadrupled but was actually decreasing when he reached his maximum RPE. In our normal volunteers (Figures 3A–C), glucose levels increased, compared to those values in the less-exhausted subjects. We want to point out, that the increase in glucose was much more prominent in our exercisers under highest RPE and more pronounced at 3,000 m than at sea level. Under hypoxia, the values in the exercisers remained elevated, whereas under normoxia, the concentrations decreased coinciding with the accumulation/formation of *ß*-hydroxybutyrate, most pronounced during recovery. Lactate and pyruvate increased with RPE in our exercising subjects (Figures 3C,D right), while the difference between oxygen levels decreased with increasing RPE. (Supplementary Figure S13) In our MPR, lactate and pyruvate concentrations showed a profile quite similar to the highly exhausted subjects. We therefore focused our attention on TCA cycle that is downstream to glycolysis and generates more ATP.

For citrate (Figure 3E), we observed similar profiles in both studies, albeit with high variability between the different subjects. Succinate and other TCA cycle intermediates (Figure 3F, Supplementary Figure S16) also increased with RPE but less at 3,000 m than at sea level. In a few selected individuals, we noted a marked increase of citrate, similar as during RPE in our MPR (Lap 3). The data suggests that not only single metabolites account for the changes in feeling state, but instead their interrelationships and ratios to one another. These observations would be in accord with those of Krebs’ findings of metabolite ratios in liver (see below), that show oxygenation status according to lactate, pyruvate and ketone-bodies. (Krebs, 1967)

We were particularly interested in ketone bodies. Acetoacetate (Figure 3G) concentrations increased both under normoxia and hypoxia, more commonly in the subjects arriving at maximal RPE. The *ß*-hydroxybutyrate values increased as well (Figure 3H), but not for the highest RPE group at 3,000 m where no increase was observed. In our MPR, acetoacetate increased already at the first time-point but decreased thereafter (Figure 3G), while in the larger subject panel at higher exhaustion acetoacetate increased and remained elevated (Figure 3G). The *ß*-hydroxybutyrate profile was similar in the MPR as in exercisers who reached a high exhaustion state at sea level but not in those under maximal RPE at 3,000 m. After the exercise session, ketone bodies accumulated as in the lower exhaustion-level groups, but to a much greater extent (16-fold) in the single MPR. In our volunteers, these values *versus* doubled in lower RPE group (Figures 3G–H).

The *a*-hydroxybutyrate concentration (Figure 3I) is a marker for early-onset insulin resistance. (Gall et al., 2010) The values increased according to RPE levels during exercise. The higher levels coincided with runs leading to elevated blood glucose levels and were already above baseline levels at the first exercise time-point in the respective runs. The general profile shape was similar in all RPE groups, while glucose and *a*-hydroxybutyrate exhibit a correlation (Supplementary Figure S17) consistent with the findings of Gall *et al*. (Gall et al., 2010) The data from our MPR underscore our technical approach. Furthermore, the data suggest that oxygen availability could be the energy-limiting factor. The fact that the glucose values increased, suggested that lack of glucose was not responsible for arriving at RPE. We therefore chose to explore energy availability. For continuous exercise, most energy is derived from oxidative-phosphorylation, thus oxygen availability is a likely highly influential factor.

We next studied the ratios of lactate/pyruvate and *ß*-hydroxybutyrate/acetoacetate which reflect the NAD^+^/NADH ratio (redox-potential) in cytosol and mitochondria respectively according to Krebs (Krebs, 1967) (Figures 3J,K). Our observations are in accordance with these ratios that were initially established in liver: in high RPE lower *Fi* O _2_ led to a more anaerobic and less aerobic metabolism, according to Krebs’ ratios. This state-of-affairs was too low to satisfy energy needs from oxidative metabolism. The TCA cycle was apparently not running as fast as necessary (in relation to glycolysis) and the NAD^+^ required for glycolysis was regenerated by lactate formation. Therefore, we observed a decrease in pH (Supplementary Figure S18). This conclusion was also underscored by the ratio of lactate/citrate. Relative lactate concentrations increased similarly in our MPR, who was able to overcome his discomfort, and in our subjects, who exerted themselves to a maximal degree to about a 4-fold increase (Figures 3C,D). The ratio of lactate/citrate increased similarly in our MPR and in this highly exerted subject group. However, in our MPR, the ratio decreased during exercise when he continued, while in the high RPE group the ratio remained. These findings suggest a higher citrate synthesis rate in our MPR after his exhaustive episode, while the recreational subjects running towards high RPE show many symptoms of metabolic acidosis as one might expect in type-1 diabetes (low pCO_2_. Low bicarbonate, low pH, and high anion gap, but also high levels of lactate, acetoacetate, and glucose). During intense exercise and low oxygen availability, we observed higher glucose levels. High lactate-pyruvate ratios (Figure 3J) and increased glucose at the same time suggest that metabolism was not able to utilize the available glucose through glycolysis and that lactate clearance was fully engaged. Integrating these results, we suggest that mitochondrial metabolism was insufficient to process the resulting pyruvate. Alanine levels that were a separator between the RPE groups (Supplementary Figure S19), coincide with increased pyruvate levels and indicate a higher reliance on the Cori and Cahill cycle.

The *a*-hydroxybutyrate (αOHB) and glucose levels were directly correlated (Figures 3B,I and Supplementary Figure S17). The αOHB concentration has been implicated as a marker sensitive to changes in glucose levels in type 2 diabetes-prone patients. (Gall et al., 2010) We observed an increase early during exercise in those subjects who later developed the highest glucose levels during exercise. Overall, the formation of lactate was similar within RPE groups; however, lactate clearance was lower at the simulated 3,000 m altitude. When similar exertional levels were achieved under different *Fi* O _2_ levels, we observed that glucose accumulated while the buffer systems in the blood were maximally challenged (Supplementary Figure S18). This observation suggests that lactate cannot be further metabolized while glucose is being funneled into the blood stream under high-energy demand conditions. Overall, empty glucose stores do not appear

To explain exhaustion, while oxygen availability and mitochondrial capacity would appear to be primarily responsible.

## 3 Discussion

We conclude that our translational experiment had utility. From single blood drops during exercising individuals, we can elucidate what is going on, better than singlularly measuring the current parameters. We believe that the most important finding in this study is that a single drop of capillary blood is useful in evaluating metabolism during exercise in contrast to metabolic studies done so far. We initially studied a serious hobby MPR, who led the way and then women and men volunteers who subjected themselves to an exercise protocol designed to address their perceived performance levels. The more strenuous exercise in terms of oxidative capacity the more glucose is used. (van Loon et al., 2001) We observed that glucose availability appeared not to be the limiting factor, but rather implicate the tricarboxylic acid→oxidative phosphorylation pathway. We were able to reduce the metabolomics dataset from a single volunteer to nine key metabolites and assessed these variables as the defining elements for the individual RPE. (Borg, 1982) The findings suggest that energy state in our setting is more dependent on oxygen than on fuel (glucose) availability. No elite athletes were represented here; however, more than 40% of marathon runners experience severe and performance-limiting depletion of physiologic reserves. The phenomenon has been attributed to carbohydrate depletion and thousands of runners drop out before reaching the finish lines. (Rapoport, 2010) This interpretation has been questioned and exercise-induced muscle damage has been suggested as being responsible. (Venhorst et al., 2018) Muscle damage can best be studied invasively; however, since exhaustion subjects recover to go on, we reasoned metabolic causes were responsible.

We did not study trained marathon or similar runners. However, we believe our findings have relevance to the personally perceived RPE value. Each and every individual must determine the exhaustion level. Although we picked the extreme RPE groups found in our dataset for mechanistic interpretation, the individuals with median RPE-levels show profiles and PCA-grouping in-between the two more extreme groups (Supplementary Figures S8–S10 and S13). Comparing samples obtained under hypoxic and normoxic conditions alone did not lead to interpretable results. Only when we grouped the samples according to RPE at final exercise time point did we find meaningful insights.

We observed known metabolic changes throughout exercise, such as an initial reliance on glucose as the main fuel source, the subsequent activity of the Cori cycle and Cahill cycle, as well as fatty-acid mobilization as indicated by increases in glycerol and free fatty acids (Figure 3A, Supplementary Figure S13). This observation makes us confident that our data reflect true metabolite behavior. The potentially novel mechanism responsible for the limit was identified by combining known facts about single metabolites and pathways, such as 2,3-BPG that changes the binding affinity of hemoglobin to blood oxygen. Thus, by not only summarily considering the orchestrated interplay of different tissues reflected in the blood metabolome, but also by considering the fact that new insights might arise from truly novel relationships, we accrued new insights. We believe that these indicators could explain why the MPR “felt badly” during exercise - most likely due to an insufficient ATP supply stemming from oxygen shortage. Succinate, which influences the carbon routing towards TCA-cycle *versus* anaerobic metabolism increased about 2-fold in our MPR, while it rose much higher in highly exhausted subjects. This state-of-affairs might indicate that in our MPR who was accustomed to exhaustion, the oxidative capacity, namely the TCA-intermediates’ basal level, was so much higher. The “mitochondrial ratio” according to Krebs shows an initial dip in both the MPR (here much stronger) and the subjects that are running towards exhaustion. However, in the MPR we observed a recovery of this ratio and, probably due to his higher oxidative capacity, the MPR was able to achieve an even higher ratio than at beginning of exercise and towards the end an even higher ratio than those subjects who were less exhausted by the exercise.

We succeeded in distilling the entire dataset of our single MPR into nine key metabolites and their interrelationships. Together, these nine metabolites accounted for four metabolic states and their three transitions. This insight was possible by the quantification of metabolites of different classes and pathways that are not measured in any clinical panel, let alone a single measurement. Erythrocyte-specific metabolites were especially crucial, as for example 2,3-BPG reflects oxygenation status. The diagnostic potential of erythrocytes is almost entirely ignored by the near exclusive investigation of serum and plasma. Although we do not have sufficient time resolution to determine in which order the switches in metabolism occur, we do have the necessary time resolution level to describe these for the first time. The observations made in our cohort under high RPE exhibited decreased pH, lowered pCO_2_ and reduced bicarbonate, while the anion gap increased (Supplementary Figure S18). The subjects’ lactate, acetoacetate, and glucose values were elevated.

TheLuebering-Rapoport shunt is a metabolic pathway in mature erythrocytes involving the formation of 2,3-bisphosphoglycerate (2,3-BPG), which regulates oxygen release from hemoglobin and delivery to tissues. 2,3-BPG, the reaction product of the Luebering-Rapoport pathway. Through the Luebering–Rapoport pathway, bisphosphoglycerate mutase catalyzes the transfer of a phosphoryl group from C1 to C2 of 1,3-BPG, giving 2,3-BPG. 2,3-bisphosphoglycerate, the most concentrated organophosphate in the erythrocyte, forms 3-PG by the action of bisphosphoglycerate phosphatase. The concentration of 2,3-BPG varies proportionately with the pH, since it is inhibitory to catalytic action of bisphosphoglyceromutase. We have strong reason to believe that this pathway played a role in our results and should be a topic of intense future investigation.

We used a metabolomics methodology that allows the quantitative determination of a large number of central metabolites and have optimized the method to allow such analysis from a single drop of full blood. Metabolomics samples were taken in combination with the recording of clinical parameters to characterize the impact of exercise on the individuals. Because full blood also includes the hematocrit, the values encompass all cellular components of the blood. As there are some substantial differences in sample handling and quenching of metabolism, we have not compared full blood against serum or plasma. Our approach quenches metabolism immediately as all cells are lysed, enzymes denatured and the extracts cooled immediately to ca. –80°C. We employed a sampling strategy that is potentially available including outside of a clinical laboratory. From our data, we conclude that we are able to detect drastic metabolic changes and that there are additional features that are exclusively measurable in whole blood. While we could not obtain absolute amounts for all metabolites, our findings rely on changes relative to baseline and on ratios of these changes. Samples of every subject under one exercise condition and at two oxygen availability level were kept in sets throughout extraction and measurement, but measurement order was randomized within the sets. Glucose and lactate, two metabolites on which are conclusions are based, were in good agreement between well-established clinical analyzers and GC–MS based measurements (Supplementary Figure S21). We determined intermediates of glycolysis and pentose phosphate pathway that were reflecting the metabolic switch when exhausted. These specific markers were only measurable from full blood during and after crisis in our MPR and were observed only in the highest intensity exercise in our second experiment. Thus they were deemed not to be general markers for RPE at the levels of analytical sensitivity we could achieve. However, the interrelationships from all measured metabolites point towards the influence of oxygen availability. The less trained subjects might have shown similar markers of oxygen release at their maximum.

There are clear limitations in our study. Our initial observations were based on a single MPR, whose fitness level was self-reported rather than measured directly. He gave a subjective RPE report and his values were measured under outdoor “field” conditions. Better would have been to test a homogeneous group of serious athletes in a common protocol to determine whether or not the results of our MPR could be repeated. Circumstances and our desire for generality dictated otherwise. We recruited a very heterogeneous group volunteers whose fitness levels were also not documented. These persons ran in a controlled setting at fixed speeds at two levels of oxygen availability. They were not required to exercise to RPE. We measured our variables in capillary blood. Our MPR was studied in the summer and our volunteer cohort was exercised at room temperatures. Under these conditions, our samples are close to, but not identical to arterial samples. Finally, we are aware that lactate kinetics, clearance, uptake, release, and turnover cannot be completely deduced from whole blood measurements. Thus, we are not able to analyze lactate as a “fulcrum of metabolism”. (Brooks, 2020)

The factor most reflected in the metabolome was RPE which in turn seems to be reflected in oxygen availability in the tissue. We suggest that exhaustion concerns an insufficiency of the tricarboxylic acid cycle and oxidative capacity. We did not begin our analysis with the goal to find reflections of RPE in the metabolome, but rather to understand the processes involved. Nevertheless, simple unsupervised data reduction technique (PCA and hierarchical clustering) reflected a connection; namely, we can perceive our metabolic status. While we refrain from postulating general biomarkers for RPE, the key to bringing metabolomics into clinical medicine is to have each person act as an own control. Longer-term observation will allow for preventive medicine and we presented here a relatively simple tool to achieve this end. Non-etheless, we now have a technology available to address these questions.

## 4 Methods

### 4.1 Study design, sample collection

#### 4.1.1 Observational study

After due procedures and written informed consent, a preliminary study was performed by a member of our laboratory. Our MPR was 26-year-old, 86 kg, 1.87 m man who views himself as competent athlete and scientist. He arrived in the laboratory at 08:00 after a 12 h overnight fast (but drank water *ad libitum*) to provoke exhaustion state, which individuals often try to ameliorate by “carbohydrate loading”. He then ran cross-country at a rate estimated <4 min/km. Each lap consisted of about 2.2 km. The few seconds necessary for sampling were accompanied by a “self-perception” RPE. (Borg, 1982) The MPR described strong exhaustion, similar to “hitting the wall”. (Rapoport, 2010) Thereafter, recovery with euphoria termed “runners’ high” has been reported. (Kozinc & Sarabon, 2017) At baseline, after each of 6 laps and after 20 min recovery, we obtained 10 µL of capillary full blood from our MPR. The samples were immediately quenched in 1 ml cold MCW (5:2:1 methanol-|chloroform|water), containing cinnamic acid as internal standard. One round, where a breakdown of performance was felt, the lap was cut short by 200 m in order not to miss this crucial observation-point. Samples were shaken and stored on dry ice. Samples were extracted as lined out below on the same day. In the framework of the subsequent study below, the Charité institutional review board allowed us to continue these investigations further. As a follow-up study, we conceived of a metabolomics study in normal volunteers.

#### 4.1.2 Prospective trial

The ethical committee of the Charité approved the study and written informed consent was obtained. The study was duly registered: ClinicalTrials.gov Identifier: NCT03121885, https://clinicaltrials.gov/ct2/show/NCT03121885 (first posted 20/04/2017). Subjects were recruited by advertisement. Men and non-pregnant women >18 years were recruited who were healthy and ingesting no medications. We purposely did not focus on fitness parameters or abilities. Athletes were not excluded but were purposely not specifically recruited. Some of the subjects were very fit and we cannot exclude the possibility that a few might have even been better than our MPR. Thirteen men and twelve women aged 18–74 years participated in the study. (ACSM et al., 2009) Please refer also to Supplementary Table S4 for an overview over subjects and their individual characterisation.

The subjects arrived in our Clinical Research Center after 12 h fasting (but drank water *ad libitum*) and underwent history and physical examinations. Body composition estimates were performed with BodPod (Life Measurement Inc. Concord, CA, United States), Bioimpedance, and a 3D Body Scanner (Human Solutions GmbH, Kaiserslautern, Germany). Venous blood was obtained for baseline, routine tests (Radiometer ABL800 Flex, Copenhagen, Denmark) and a resting electrocardiogram was performed. Blood pressure was measured oscillometrically and anthropometric data were obtained. The subjects were questioned as to exercise habits and rendered an assessment of their fitness levels.

We determined the performance levels, (Jones et al., 1985), as adapted to treadmill exercise according to normal standards as estimated from ergometer testing indoor. We aimed for an estimated eight metabolic equivalent of task (MET) performance for 30 min. If this task was insufficient to exhaust the subjects, the test was repeated with a 30% increment and in some very fit individuals a 60% increment was performed. To determine RPE, we relied on a 1-through-10 modified Borg scale. (Borg, 1982) Subjects were randomized to order of exercise at sea level (*Fi* O _2_ 20.9%) or to normobaric hypoxia (altitude 3,000 m, *Fi* O _2_ 14.5%). They were unaware of the regimens provided, as our chamber was used for all studies. Respective to performance-ability, baseline, 10 min, 20 min, and 30 min samples (or when RPE was experienced) as well as 10 min (recovery 1) and 20 min (recovery 2) after exercise of 20 µL capillary full blood was taken from the ear-lobe and immediately quenched in 1 ml cold MCW (5:2:1 methanol|chloroform|water) containing cinnamic acid as internal standard, shaken, and stored on dry ice Samples were stored at –80°C until further extraction.

At blood drawing, subjects were asked to estimate their performance stress on a scale of 1–10, similar to the one established by Borg. (Borg, 1982) Samples were collected in ice cold methanol | chloroform | water (5:2:1) containing cinnamic acid, immediately quenching metabolic activity. Capillary blood 10 µL from the earlobe was obtained at baseline, at 30 min or at exhaustion, and 30 min after exercise. In these samples, we measured blood gases for pH, pO_2_, pCO_2_, $HCO_{3}^{-}$ , Na^+^, K^+^, Cl^−^, Ca^2+^, and anion gap (Radiometer). Glucose and lactate were measured separately with routine chemical analysis. Blood pressure, heart rate, and pulse oximetry were determined at baseline, 10 min, 20 min, 30 min or at exhaustion, and 20 min and 30 min of recovery.

### 4.2 Metabolomics

#### 4.2.1 Sample extraction

Samples were removed from the freezer in batches and kept at 4°C throughout the extraction process. 500 µL water (in the prospective trial also containing isotopically labeled internal standards for normalization) were added to induce phase separation. Samples were shaken (Eppendorf 1,000 rpm) for 20 min to ensure phase equilibration. After 10 min centrifugation, polar (upper) and lipid phase (lower) were obtained. Lipid extracts were dried under nitrogen stream and stored at –80°C until measurement. Polar phase extracts were dried in a rotational vacuum concentrator (Martin Christ, Germany) without heating in <4 h. Samples were stored at –80°C (observational study, –20°C) until derivatization.

#### 4.2.2 Standardization

For substance identification across batches, we used mixtures of 100 substances to compare RI and mass spectra. For 69 substances we measured 8-point calibration curves to check linearity (Pietzke et al., 2014). Sample intensities within one set of experiments were standardized by cinnamic acid added to the extraction solvent (MCW). Furthermore, we added 2 stably labeled isotopomers during extraction (see also Quantification/Normalization). As such, we standardized by cinnamic acid and used fully labeled lactate and glucose to assess our quantification against the established clinical methods. (Supplementary Figure S21)

#### 4.2.3 Derivatization

For derivatization, extracts were thawed in a rotational vacuum concentrator (Martin Christ, Germany) without heating for 20 min 10 µL of 40 mg methoxyamine hydrochloride/mL pyridine were added, samples were incubated for 90 min at 30°C. Next 30 µL of MSTFA containing 200 μg/ml *n*-alkanes (C_10_, C_12_, C_15_, C_17_, C_19_, C_22_, C_28_, C_32_, C_36_) as retention index markers were added as previously described (Pietzke et al., 2014). Derivatization was carried out simultaneously for every sample in a single measurement batch.

#### 4.2.4 Randomization

Samples were randomized as follows: To ensure highest level of comparability among one subject’s samples blocks from one subject’s performances at a certain exercise intensity at different *Fi* O _2_ were formed giving blocks of ≤12 samples (2 *Fi* O _2_ levels and maximum 6 time-points, depending on ability). These blocks were kept throughout extraction, derivatization and measurement. Extraction batches (*n* = 10) consisted of ≤4 blocks, measurement batches (*n* = 10) also contained ≤4 blocks but consisted of different sets of randomly selected blocks. Measurement order within one block was randomized.

#### 4.2.5 Gas chromatography-mass spectrometry measurement

Gas chromatography-mass spectrometry was carried out using a previously published method using a Pegasus IV GC-ToF MS (Leco, United States) (Pietzke et al., 2014). Scan rates of 20 Hz and a mass range of 70–600 Th were used. Ionization energy was set to 70 eV. Gas chromatographic separation of compounds was performed on an Agilent 6890N (Agilent, Santa Clara, CA, United States) equipped with a VF-5ms column of 30 m length (Varian, Palo Alto, CA, United States). The initial temperature was held at 67.5°C for 2°min, followed by a temperature gradient of 5°C min^−1^ until 120°C, then 7°C min^−1^ until 200°C, followed by 12°C min^−1^ until 320°C with a hold time of 6 min. The transfer line was kept at 250°C throughout. A cold injection system was used with a matching baffled deactivated liner (CIS4, Gerstel, Mülheim an der Ruhr, Germany), operating in split mode (split 1:5, injection volume 1 μL), with the following temperature gradient applied: hold of the initial temperature of 80°C for 0.25°min, followed by a temperature increase of 12°C s^−1^ to 120°C, followed by a temperature increase of 7°C s^−1^ to 300°C with a hold time of 2 min.

#### 4.2.6 Peak picking, annotation

Data was smoothed and baseline corrected using ChromaTOF (vendor software). Peaks were picked using ChromaTOF with a signal to noise threshold of 20. Given the rather small sample size, formal tests for Gaussian distribution and linearity would be underpowered and not informative. Accordingly, we decided to use Spearman rank correlations to produce the correlation matrix underlying the PCA.

Annotation was performed using an in-house version of a published software (Kuich et al., 2014), as well as using manual inspection with proprietary software (ChromaTOF, LECO). This approach allowed us to inspect the mass spectra of each peak from all measurements individually. We matched peaks stepwise against i) standard mixes included at the beginning of every batch (library size = 137) (Opialla et al., 2020), as well as ii) an in-house library (library size = 12) of compounds individually measured on our machines, and iii) a subset of the Golm-metabolome database (Kopka et al., 2005). For the observational study, we also annotated and reported unidentified but consistently occurring peaks. Lipid compounds were matched against an in-house library (library size = 36).

#### 4.2.7 Quantification, normalization

Metabolites were quantified using the top 5 mass traces according to intensity, excluding masses if adjacent peaks had same nominal mass and mass traces originating from derivatization agents (e.g. 73 Th, 147 Th). Also, characteristic masses were included purposefully (e.g. 299 Th for phosphates). The scans along the peaks were summed up to give AUC without interpolation. Glucose and lactate were also measured with clinically approved methods, so we used the included u-^13^C-labeled substances added during extraction, to compare clinical measurements, our top-5 approach and the current gold standard: heavy labeled internal standards. For glucose we used the ion pairs 319/323 Th and 217/220 Th, for lactate 117/119 Th and 190/193 Th. Samples were normalized using cinnamic acid included in the extraction solvent at sample collection.

#### 4.2.8 Missing values

As with any MS-dataset, several metabolites have missing values. Except for clear oxygenation markers accordingly with *Fi* O _2_ and effort level (ribose-5-phosphate and ribulose-5-phosphate, Supplementary Figures S22A, 23) and iso-aminobutyrate in females (Supplementary Figure S22B), no compound was significantly missing more in one condition. After careful manual curation we found, that with generally lower intensity also more missing values occur (NMAR). We therefore treated the missing values as not missing at random values (NMAR). Values were imputed using QRILC (Lazaar, 2015) on a per metabolite basis on the normalized values, we allowed generally up to 20% missing values. If fraction of missing values was higher, metabolites were excluded from multivariate statistics in the prospective trial.

#### 4.2.9 Statistical analyses, time-profiles

Statistical analysis was carried out using R and tidyverse (https:// www.tidyverse.org/packages); visualizations except where noted, were created using ggplot2 and inkscape (https://inkscape.org/release/inkscape-0.92.4).

Since some of the subjects in the prospective trial were not able to complete the exercise bout (resulting in very low RPE values) we re-encoded data collected at last exercise time-point as 30 min exercise value. As not every individual was able to complete the exercise, we sometimes obtained less than three samples from exercise. For plotting time-profiles we shifted the samples in time in a way that all values obtained from final exercise time-point have the same time coordinate, as this reflects the most similar state possible, when dealing with such a heterogeneous group of performance levels as in our study. Normally distributed clinical data were statistically analyzed by repeated-measures analysis of variance with appropriate adjustments. For PCA we removed outliers according to Hotelling’s-*T* criteria. For line-plots all samples were included. From a statistics point of view, principal components are unobservable higher-order traits covering a wider range of observable measures. Naming those components is inevitably arbitrary, and we deduced the main shared feature from the underlying highly correlated traits.

The authors confirm that all methods were carried out in accordance with relevant guidelines and regulations*.*

## Data Availability

The original contributions presented in the study are included in the article/Supplementary Material, further inquiries can be directed to the corresponding authors.
