# Corrigendum: Exercise blood-drop metabolic profiling links metabolism with perceived exertion

**DOI:** 10.3389/fmolb.2023.1129602

**Published:** 2023-03-10

**Authors:** Tobias Opialla, Benjamin Gollasch, Peter H. J. L. Kuich, Lars Klug, Gabriele Rahn, Andreas Busjahn, Simone Spuler, Michael Boschmann, Jennifer A. Kirwan, Friedrich C. Luft, Stefan Kempa

**Affiliations:** ^1^ Department of Proteomics and Metabolomics Max-Delbrück-Center for Molecular Medicine Berlin, Berlin Institute for Medical Systems Biology, Berlin, Germany; ^2^ Muscle Research Unit, Experimental and Clinical Research Center, A Joint Collaboration Between Max-Delbr ück-Center and Charité Universitätsmedizin Berlin, Berlin, Germany; ^3^ Berlin Institute of Health Metabolomics Platform, Charite Universitätsmedizin Berlin, Berlin, Germany; ^4^ Experimental and Clinical Research Unit, Joint collaboration between Max-Delbr ück-Center and Charité Universitätsmedizin Berlin, Berlin, Germany; ^5^ HealthTwiSt GmbH, Berlin, Germany

**Keywords:** gas chromatography, blood drop sampling, relative perceived exertion, hypoxia, metabolomics

In the **Abstract** Section, **Methods**, Paragraph Number 1 of the original article, there was an error. This sentence previously stated appears below:

“We first observed a single volunteer who ran 13 km in 60 min”

The corrected sentence is:

“We first observed a single volunteer who ran 13 km in 61 min”

The authors apologize for this error and state that this does not change the scientific conclusions of the article in any way. The original article has been updated.

