# Supplementary material for: Exercise blood-drop metabolic profiling links metabolism with perceived exertion
Source: Front Mol Biosci. 2022 Dec 21;9:1042231. doi: 10.3389/fmolb.2022.1042231 (PMC9822726; doi:10.3389/fmolb.2022.1042231)
Supplement: Supplementary file 2 [file Table1.DOCX]

# Supplement One Drop

## Table S1 Track run and lap times

| **Lap** | ***t* 1/min** | **Distance 1/km** |
| --- | --- | --- |
| L1 | 10 | 2.2 |
| L2 | 10 | 2.2 |
| L3 | 13 | 2.0 |
| L4 | 8 | 2.2 |
| L5 | 10 | 2.2 |
| L6 | 10 | 2.2 |


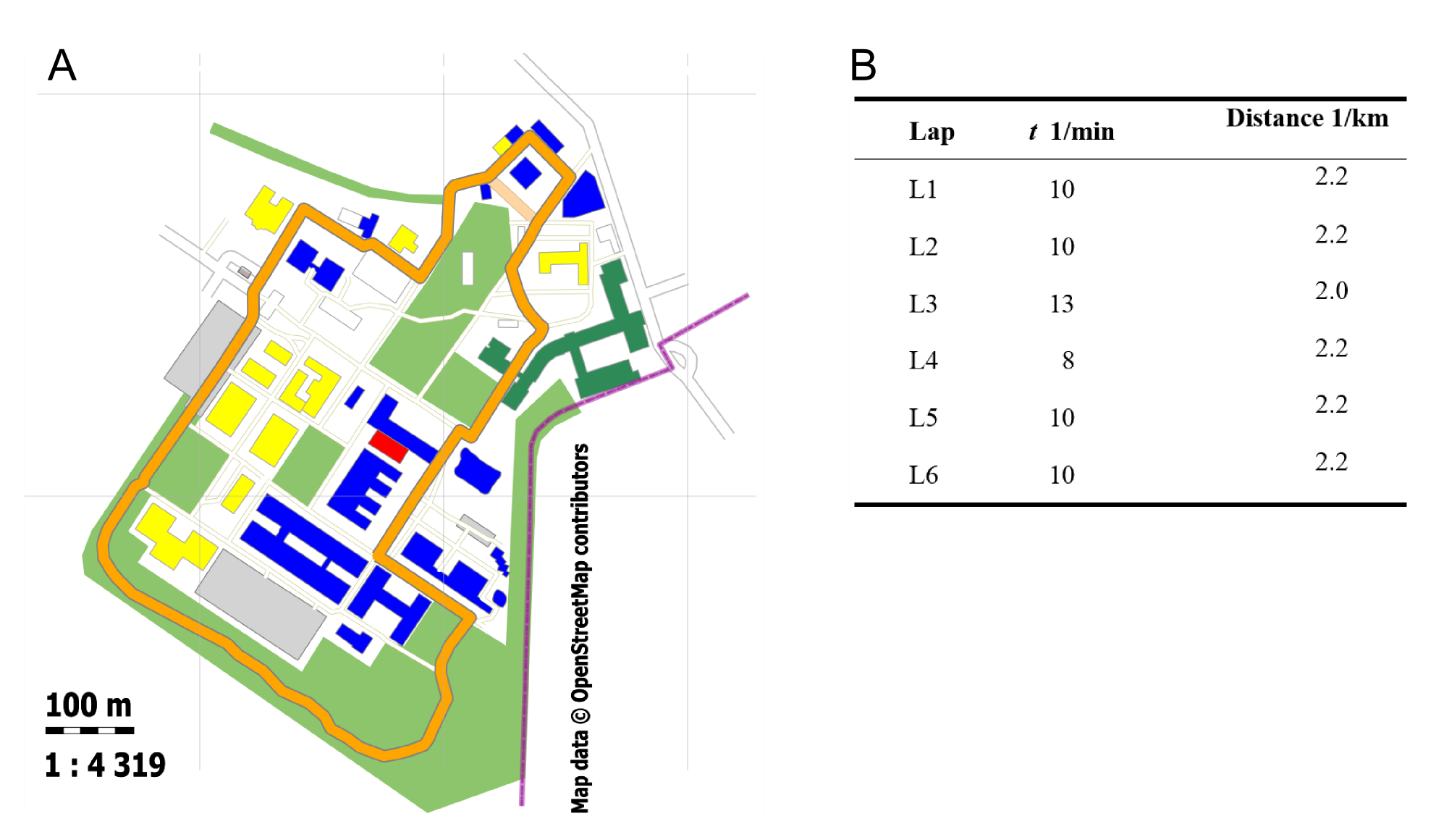
Table S1 related to figure 1, Map, distance, and lap times of the run

B

(A) The track is colored in orange, the shortcut mentioned in the methods in L3 in light orange at the top right. (Scale 1:4319, generated with OpenStreetMap (http://www.openstreetmap.org/)).

(B) A table showing the distance and corresponding time for each lap completed by the volunteer. Most notably, the lap during which “hitting the wall” (L3) was experienced, although shorter by 200 m, was the slowest lap of the run. In contrast, the lap during which the “high” was experienced (L4) was the fastest.

## Table S2 Substances quantified in single volunteer

Table S2 Related to figure 1 List of Metabolites identified from Metabolomics measurements across all samples of our single volunteer.

| Acetoacetate | Glucuronate | Nonanoate |
| --- | --- | --- |
| Alanine | Glutamate | Octadecadienoate, 9,12-(Z,Z)- |
| Alanine, beta- | Glutarate, 2-hydroxy- | Octadecanoate |
| Arabitol | Glutarate, 2-oxo- | Octadecenoate, 9-(E)- |
| Aspartate | Glycerate | Octadecenoate, 9-(Z)- |
| Benzoate | Glycerate-2,3-bisphosphate | Octanoate |
| Butanoate, 2-amino- | Glycerate-3-phosphate | Ornithine |
| Butanoate, 2-hydroxy- | Glycerol | Phenylalanine |
| Butanoate, 3-hydroxy- | Glycerol-3-phosphate | Phosphoenolpyruvate |
| Cholesterol | Glycerophosphoglycerol | Proline |
| Citrate | Glycine | Pyridine, 2-hydroxy- |
| Creatinine | Glycolate | Pyroglutamate |
| Decanoate | Guanosine-5-monophosphate | Pyruvate |
| Dehydroascorbate, dimer | Gulonate | Ribose-5-phosphate |
| Docosahexaenoate, 4,7,10,13,16,19-(Z,Z,Z,Z,Z,Z)- | Heptadecanoate | Ribulose-5-phosphate |
| Docosanoate | Hexadecanoate | Serine |
| Docosatetraenoate, 7,10,13,16-(Z,Z,Z,Z)- | Hexadecenoate, 9-(Z)- | Sorbitol |
| Dodecanoate | Hexanoate | Succinate |
| Eicosanoate | Hypotaurine | Sucrose |
| Eicosatetraenoate, 5,8,11,14-(Z,Z,Z,Z)- | Inositol, myo- | Tetradecanoate |
| Erythritol | Isobutanoate, 3-amino- | Threonate |
| Ethanolamine | Isoleucine | Threonine |
| Ethanolaminephosphate | Lactate | Tricosanoate |
| Fructose | Lactose | Triethanolamine |
| Fructose-6-phosphate | Leucine | Tryptophan |
| Fumarate | Lysine | Tyrosine |
| Galactose | Malate | Uracil |
| Gluconate | Maltose | Urate |
| Glucosamine, N-acetyl- | Mannitol | Urea |
| Glucose | Methionine | Uridine |
| Glucose-6-phosphate (or Glucose-1-phosphate) | Nicotinamide | Valine |

## Figure S1 Metabolic Map of Single Volunteer


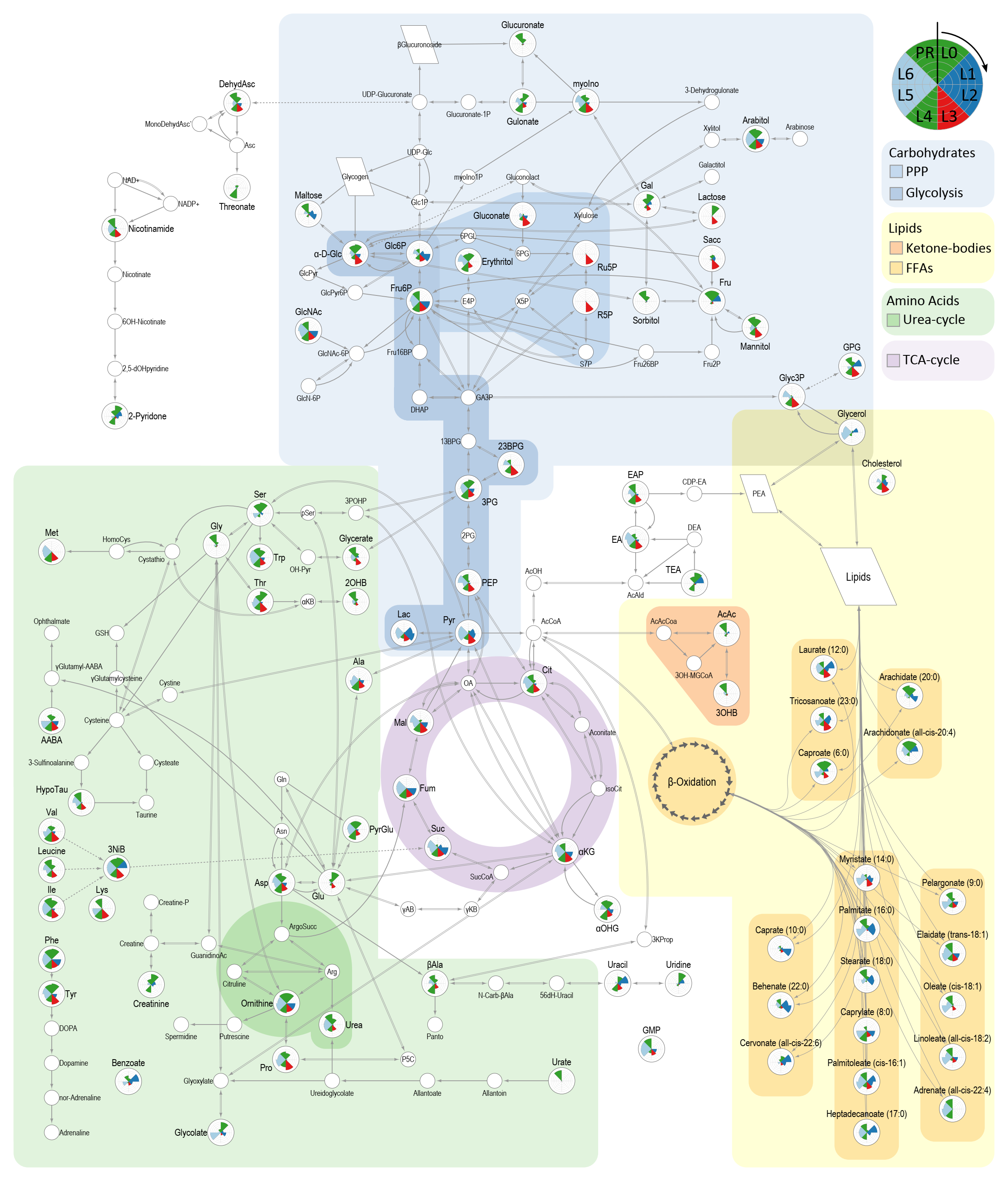


Figure S1 Related to figure 1, Metabolic network showing profiles of all metabolites identified across the exercise bout of a single volunteer. Colors according to metabolic phase, height of each piece: z-scored fold change whithin each metabolite.

## Table S3 Demographics of Study panel

Table S3 Related to figure 2 Demographic variables of the volunteer subjects
(Quantitative variables ± standard deviation (SD), or fraction in braces

| Variable | Value |
| --- | --- |
| Age mean (range) | 38 (27…74) years |
| Sex | Women: 10 (38%)  Men: 16 (62%) |
| Dietary preferences | Vegetarian: 4 (15%)  Vegan: 1 (4%)  Omnivorous: 21 (81%) |
| Cigarette Smoking | Smoker: 1 (4%)  Non-smoker: 25 (96%) |
| Perceived fitness training level | Rarely: 1 (4%)  1-2/week: 6 (23%)  2-3/week: 10 (38%)  3-4/week: 6 (23%)  1>5/week: 3 (12%) |
| Body mass index | 23.1±2.6 |
| Systolic blood pressure | 126±13 mmHg |
| Diastolic blood pressure | 78±12 mmHg |
| Resting heart rate | 61 beats/min |

## Table S4 Performance overview

Table S4 , related to figure 2, Supplemental Table of individual Subjects’ performance

| **Subject** | **Sex** | **Age** | **Fitness Level** | **Height** | **Body Mass** | **Sport per Week** | **Nutrition** | **Effort level** | | | | | | | | | | |
| --- | --- | --- | --- | --- | --- | --- | --- | --- | --- | --- | --- | --- | --- | --- | --- | --- | --- | --- |
|  |  | 1/y | self rated (1…10) | 1/cm | 1/kg |  |  | Jones "normal" | | |  | Jones level +30% | | |  | Jones level +60% | | |
|  |  |  |  |  |  |  |  | ***V*** (1/(km/h)) | **Normoxia** | **Hypoxia** |  | ***V*** (1/(km/h)) | **Normoxia** | **Hypoxia** |  | ***V*** (1/(km/h)) | **Normoxia** | **Hypoxia** |
| Q | **♀** | **23** | **5** | 168 | 59.8 | **rarely** | omnivorous | 8.3 | **30** | **16** |  |  |  |  |  |  |  |  |
| O | **♀** | **26** | **4** | 160 | 60.8 | **1-2/week** | omnivorous | 6.6 | **30** | **25** |  |  |  |  |  |  |  |  |
| F | **♀** | **26** | **8** | 167 | 57.3 | **3-4/week** | vegetarian | 8.2 | **30** | **30** |  |  |  |  |  | 13.3 | **30** | **10** |
| L | **♀** | **27** | **7** | 170 | 60.8 | **3-4/week** | vegetarian | 8.2 | **30** | **30** |  |  |  |  |  |  |  |  |
| A | **♀** | **28** | **6** | 160 | 47.6 | **2-3/week** | omnivorous | 8.6 | **30** | **30** |  | 11.1 | **30** | **26** |  |  |  |  |
| H | **♀** | **31** | **6** | 170.5 | 73.1 | **2-3/week** | omnivorous | 6.9 | **30** | **30** |  |  |  |  |  |  |  |  |
| Y | **♀** | **39** | **7** | 162 | 68.49 | **2-3/week** | omnivorous |  |  |  |  | 7.1 | **30** | **16** |  |  |  |  |
| X | **♀** | **46** | **8** | 169.9 | 70.9 | **2-3/week** | omnivorous |  |  |  |  | 7.5 | **30** | **30** |  |  |  |  |
| J | **♀** | **51** | **6** | 176 | 62.5 | **2-3/week** | omnivorous | 7.3 | **30** | **30** |  |  |  |  |  |  |  |  |
| S | **♀** | **54** | **8** | 168 | 61.7 | **>5/week** | omnivorous |  |  |  |  | 7.8 | **30** | **15** |  |  |  |  |
| G | **♂** | **18** | **9** | 181 | 62.9 | **3-4/week** | omnivorous | 12.2 | **30** | **30** |  | 15.9 | **27** | **15** |  |  |  |  |
| K | **♂** | **21** | **8** | 184 | 88.5 | **3-4/week** | omnivorous | 8.7 | **30** | **30** |  | 11.3 | **30** | **27** |  | 13.7 | **30** | **16** |
| D | **♂** | **25** | **8** | 180 | 76.2 | **2-3/week** | omnivorous | 9.5 | **30** | **30** |  | 12.4 | **30** | **28** |  |  |  |  |
| E | **♂** | **28** | **6** | 188 | 79.8 | **1-2/week** | omnivorous | 9.8 | **30** | **30** |  | 12.7 | **20** | **13** |  |  |  |  |
| C | **♂** | **29** | **7** | 175 | 80.9 | **3-4/week** | omnivorous | 8.1 | **30** | **30** |  | 10.5 | **30** | **30** |  | 10.5 | **30** | **30** |
| M | **♂** | **32** | **7** | 178 | 82.7 | **2-3/week** | omnivorous |  |  |  |  | 10.5 | **30** | **30** |  | 12.9 | **30** | **16** |
| R | **♂** | **36** | **10** | 176 | 60.0 | **>5/week** | vegetarian |  |  |  |  | 14.0 | **30** | **30** |  |  |  |  |
| N | **♂** | **46** | **8** | 179 | 68.8 | **2-3/week** | omnivorous | 9.3 | **30** | **30** |  | 12.1 | **30** | **30** |  |  |  |  |
| P | **♂** | **50** | **7** | 182 | 70.3 | **3-4/week** | omnivorous |  |  |  |  | 12.0 | **30** | **15** |  |  |  |  |
| U | **♂** | **51** | **6** | 186 | 72.3 | **2-3/week** | vegan | 9.5 | **30** | **16** |  |  |  |  |  |  |  |  |
| I | **♂** | **52** | **7** | 168 | 76.5 | **1-2/week** | omnivorous | 6.5 | **30** | **30** |  | 8.5 | **30** | **30** |  |  |  |  |
| B | **♂** | **53** | **4** | 179 | 77.6 | **1-2/week** | omnivorous | 7.7 | **30** | **30** |  | 10.0 | **30** | **20** |  |  |  |  |
| T | **♂** | **55** | **3** | 187 | 85.2 | **1-2/week** | vegetarian | 7.7 | **30** | **30** |  |  |  |  |  |  |  |  |
| W | **♂** | **55** | **6** | 180 | 75.8 | **2-3/week** | omnivorous | 7.9 | **30** | **16** |  |  |  |  |  |  |  |  |
| V | **♂** | **55** | **8** | 179 | 81.6 | **1-2/week** | omnivorous |  |  |  |  | 9.3 | **30** | **30** |  |  |  |  |
| Z | **♂** | **74** | **8** | 179 | 85.1 | **>5/week** | omnivorous |  |  |  |  | 8.6 | **30** | **30** |  |  |  |  |

Overview of the individuals in our panel of recreational runners. Most are lean, ominivorous and moderately physically active. Ages span all age groups, with a slight tendency to middle aged persons.

## Table S5 Statistics of “Clinical” measurements

Table S5 Related to figure 2B, Change across all conditions: Effects of exercise on changes in variables from resting to maximum exertion at sea level (A), 3000 m (B), and exercise levels (C). Due to lower n-numbers, running at 1.6× normal speed was not used in the analysis. Anion gap (AG), glucose, HCO_3_ and lactate are given in mmol/L. Heart rate (HR) beats per minute, O_2_ content mL per L whole blood.

Table S5A Difference between rest and exercise at sea level.

| *Variable* | *Last exercise*  *delta_to baseline* | *p* | *2^nd^ Recovery delta to baseline* | *p* |
| --- | --- | --- | --- | --- |
| AG delta | 2.0 (1.0/5.6) | 0.001 | 1.0 (0.0/3.0) | 0.001 |
| Glucose delta | 7 (-1/27) | 0.001 | -3 (-6/4) | 0.399 |
| HCO_3_ delta | -1.9 (-6.1/-0.5) | 0.001 | -0.4 (-1.4/0.0) | 0.001 |
| HR delta | 93 (77/110) | 0.001 | 17 (10/23) | 0.001 |
| Lactate delta | 1.9 (0.8/6.7) | 0.001 | 0.3 (0.1/1.4) | 0.001 |
| pCO_2_ delta | -3.0 (-5.6/-1.4) | 0.001 | -1.2 (-2.3/-0.1) | 0.001 |
| pH delta | -0.014 (-0.091/0.001) | 0.001 | -0.001 (-0.018/0.008) | 0.165 |
| RPE delta | 3.0 (6.0/3.0) | 0.001 | 0 (0/0) | NaN |
| Oxygen content delta | -0.05 (-0.18/0.16) | 0.623 | -0.13 (-0.28/-0.01) | 0.001 |

Table S5B Difference between exercise at sea leavel and exercise at 3000 m, values are relative to baseline.

| *Variable* | *Last exercise delta_to baseline*  *at Sea level* | *at 3000 m* | *p* |
| --- | --- | --- | --- |
| AG delta | 1.0 (0.0/3.0) | 4.0 (2.0/7.0) | 0.005 |
| Glucose delta | 4 (-2/14) | 13 (-0/47) | 0.024 |
| HCO_3_ delta | -1.3 (-2.2/-0.1) | -4.9 (-7.6/-1.1) | <0.001 |
| HR delta | 92 (71/108) | 94 (82/114) | 0.412 |
| Lactate delta | 1.1 (0.4/2.4) | 4.2 (1.4/8.0) | 0.001 |
| pCO_2_ delta | -2.4 (-4.2/-1.5) | -4.9 (-6.2/-1.4) | 0.093 |
| pH delta | -0.001 (-0.023/0.008) | -0.059 (-0.113/-0.012) | <0.001 |
| RPE delta | 3.0 (5.0/2.0) | 5.0 (6.0/3.0) | 0.040 |
| Oxygen content delta | -0.07 (-0.15/0.13) | 0.02 (-0.18/0.25) | 0.403 |

Table S5C Difference in change between baseline and final exercise timepoint for different exercise intensity levels (standard intensity according to Jones (Jones et al., 1985) and 30% increase of running speed).

| *Variable* | *Last exercise delta_to baseline*  *at normal speed* | *at 1.3× normal speed* | *p* |
| --- | --- | --- | --- |
| AG delta | 2.0 (0.0/3.3) | 4.0 (1.0/7.0) | 0.011 |
| Glucose delta | 4 (-4/12) | 14 (1/45) | 0.007 |
| HCO_3_ delta | -0.8 (-3.0/-0.1) | -4.5 (-8.1/-1.5) | <0.001 |
| HR delta | 80 (70/97) | 102 (93/119) | <0.001 |
| Lactate delta | 1.0 (0.4/3.1) | 4.7 (1.4/8.7) | <0.001 |
| pCO2 delta | -2.4 (-3.7/-0.8) | -5.4 (-6.9/-2.4) | <0.001 |
| pH delta | -0.007 (-0.023/0.006) | -0.048 (-0.115/-0.010) | 0.002 |
| RPE delta | 3.0 (4.3/2.0) | 6.0 (6.0/3.2) | 0.001 |
| Oxygen content delta | -0.00 (-0.17/0.14) | -0.08 (-0.20/0.17) | 0.884 |

## Figure S2 Correlation of Clinical measures


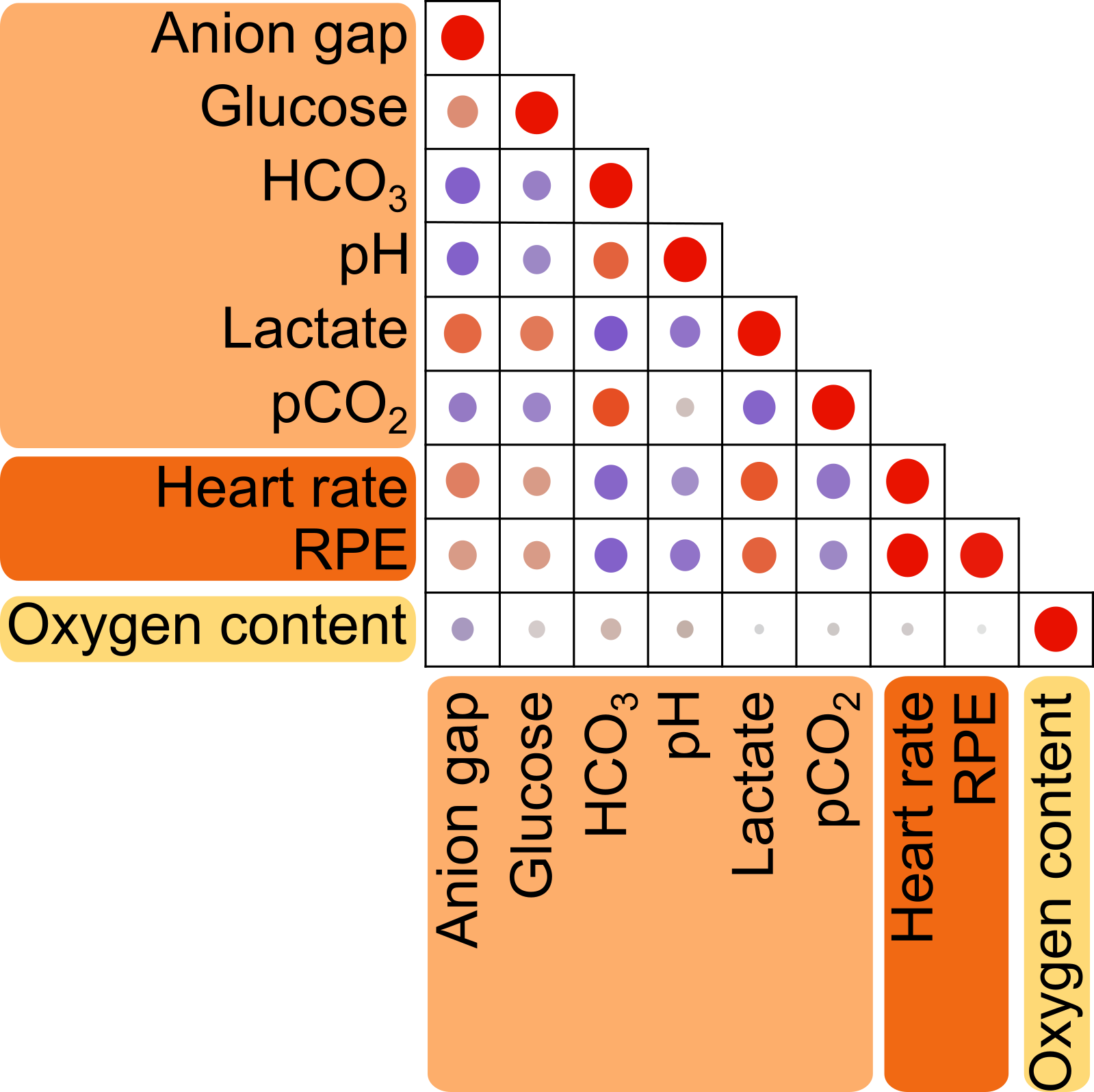


Figure S2 Related to Figure 2B, Principal component analysis (PCA) of clinical measurements.

The correlation coefficients of individual variables to the three components found (Figure 2B) are shown. Exhaustion was comprised principally from the arterial blood gas variables, lactate, and degree of metabolic acidosis (anion gap). Exertion was explained by heart-rate changes and rate of perceived exertion (RPE). Oxygen content was a single factor; this component remained flat during exercise.

## Figure S3 individual exhaustion profiles
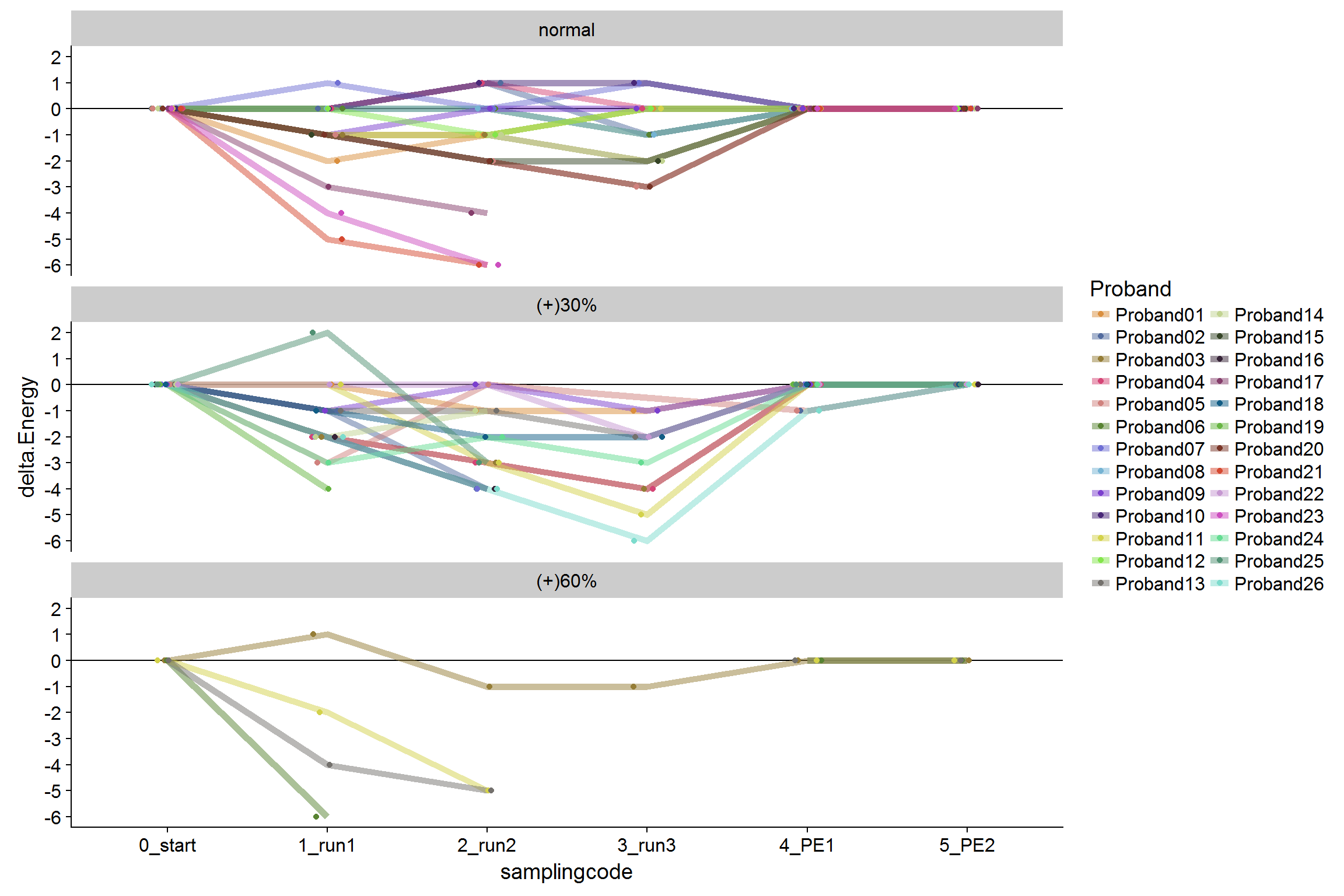


Figure S3 Related to Figure 2E, Except for a few, hypoxia showed very little influence on overall energetic feeling in normal conditions, sometimes performing at hypoxia was even perceived less exhaustive throughout the run. At 30% and 60% increase, hypoxia was perceived as more strenuous than normoxia.

##
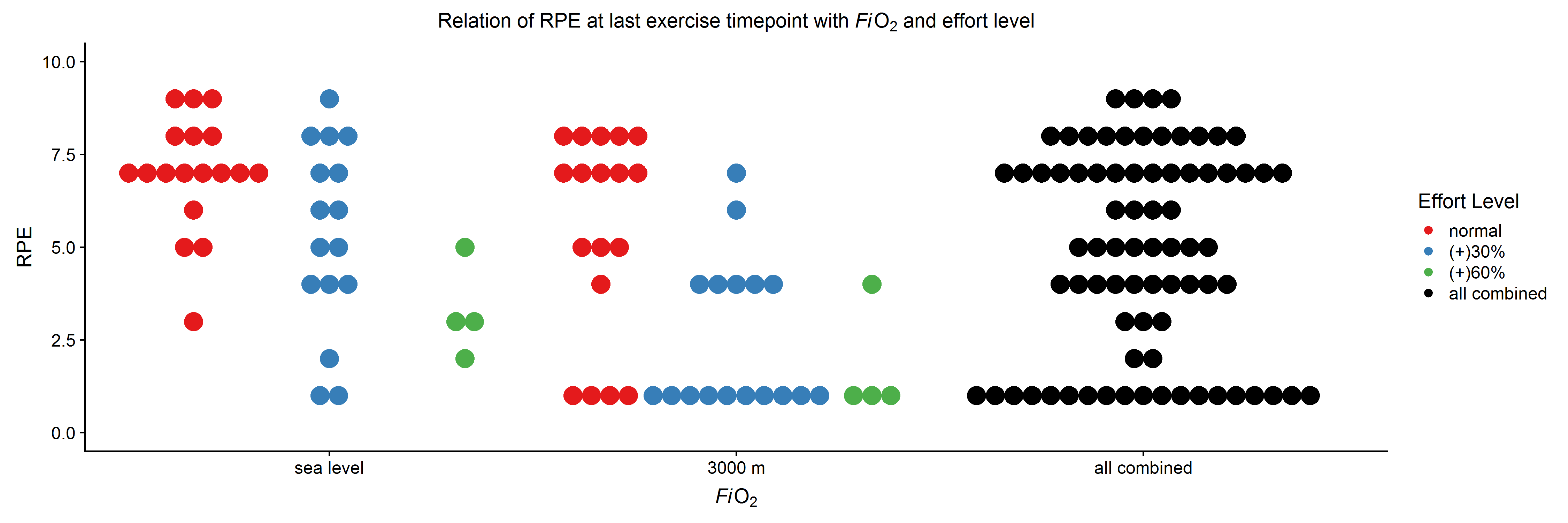
Figure S4 Distribution of RPE at last exercise time point

Figure S4 Related to Figure 2F, Distribution of ratings of perceived exertion at last exercise timepoint, grouped by running speed (effort level, color coding) and FiO2. There is a slight dependence of the combination of running speeds and FiO2, obviously since running at higher speed and less oxygen is more exhausting. But there is no significant trend according to just one of the parameters defining the challenge.

## Figure S5 PCA Metabolite measurements


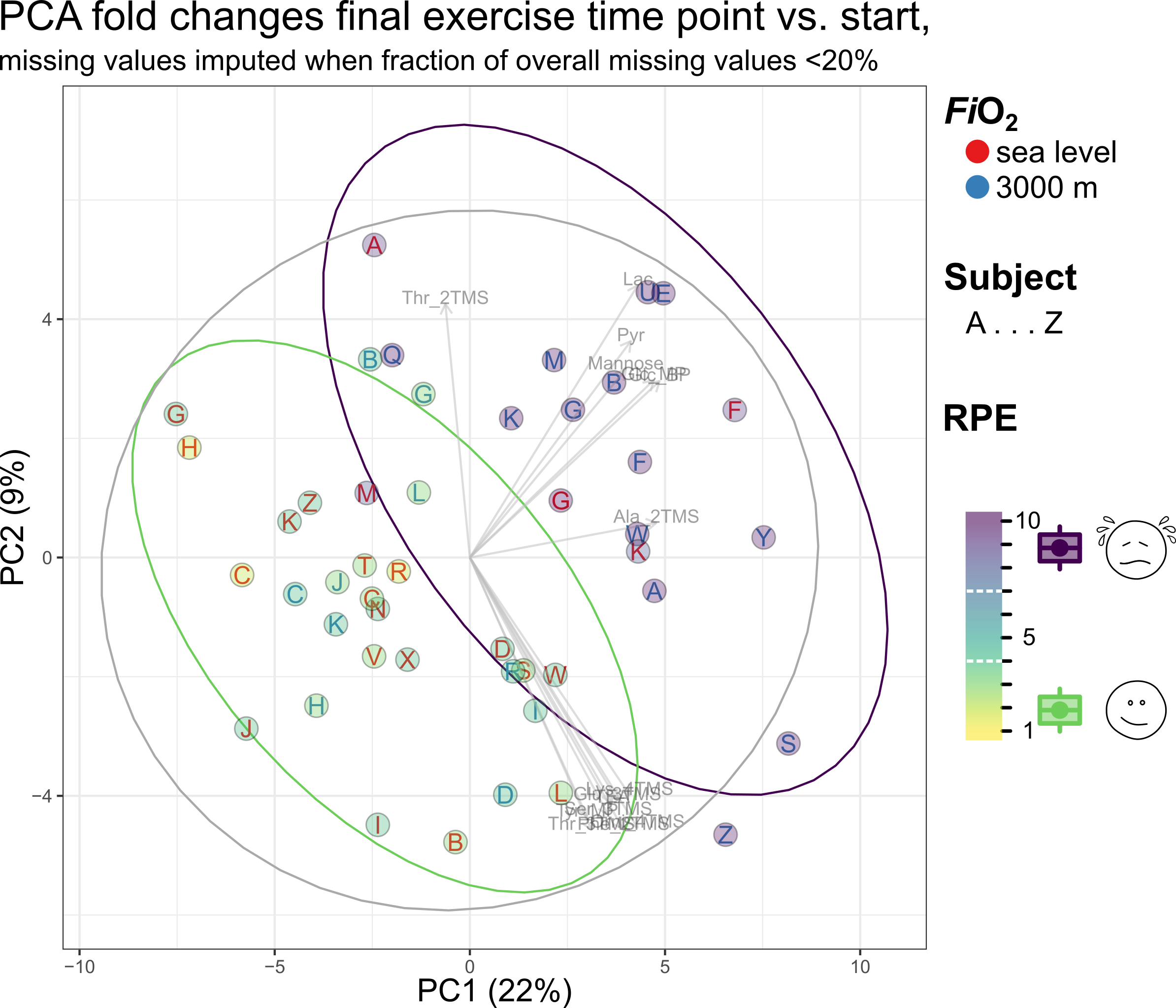


Figure S5 Related to figure 2G-I, PCA of fold change of metabolite intensities from baseline to final exercise time point including only subjects arriving in one of the two more extreme groups forming in the RPE levels. Pointareas are shaded by RPE, letters depicting individuals are colored according to oxygenation state. Extreme outliers (not shown) according to the T-ellipse (gray) were removed, and PCA was re-run.

## Figure S6 Dogleg plot of contributors for separation between confidence ellipese’s centers


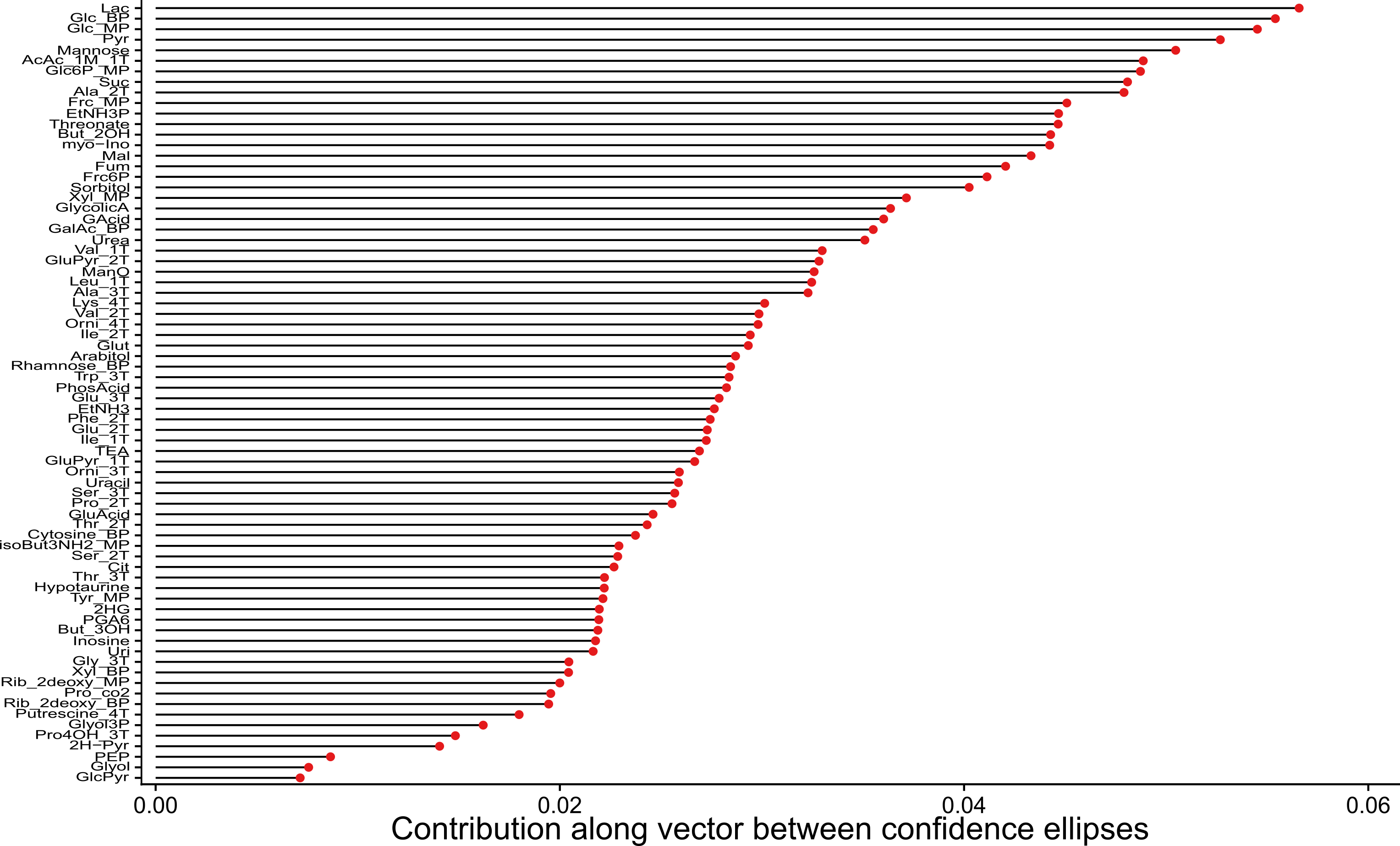


Figure S6 Related to figure 2G-I, Contributions of individual metabolites to separation between confidence ellipse centers, calculated from loadings of PC1 and PC2 of a PCA, performed for members of the more extreme RPE-groups. The most influential metabolites are discussed in the main text, Glucose has a high influence as well as alanine, mannose, lactate and pyruvate, indicating a highly active Cahill-cycle.

##
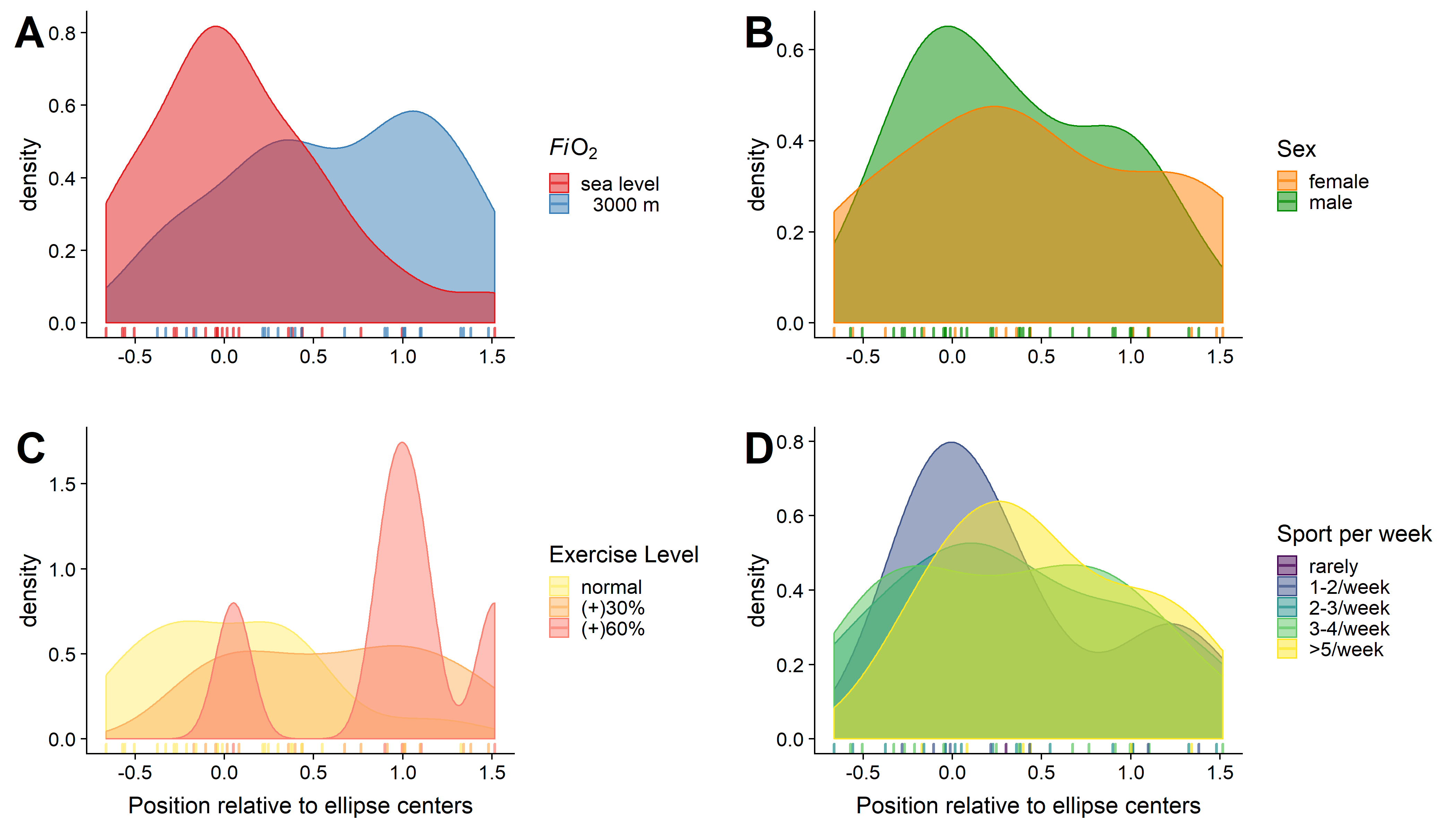
Figure S7 Distribution of phenotypes and experimental conditions alongs ellipses centers

Figure S7 Related to figure 2G-I, Distributions of samples along the confidence ellipse centers connecting vector from PCA above (only extreme RPE groups), according to FiO_2_ (A), sex (B), exercise level (C) and times per week sport is performed (D) by the recreational athletes (not assessing physical activity quality or duration). The strongest separation is by RPE (figure 2G), although some separation according to FiO_2_ is and exercise level is also observed. When all RPE-groups are considered (Figure S12) this pattern decreases: it is likely that we just see, that hypoxia and high exercise intensity facilitate arrival at high RPE.

## Figure S8 PCA Metabolite measurements including middle RPE group


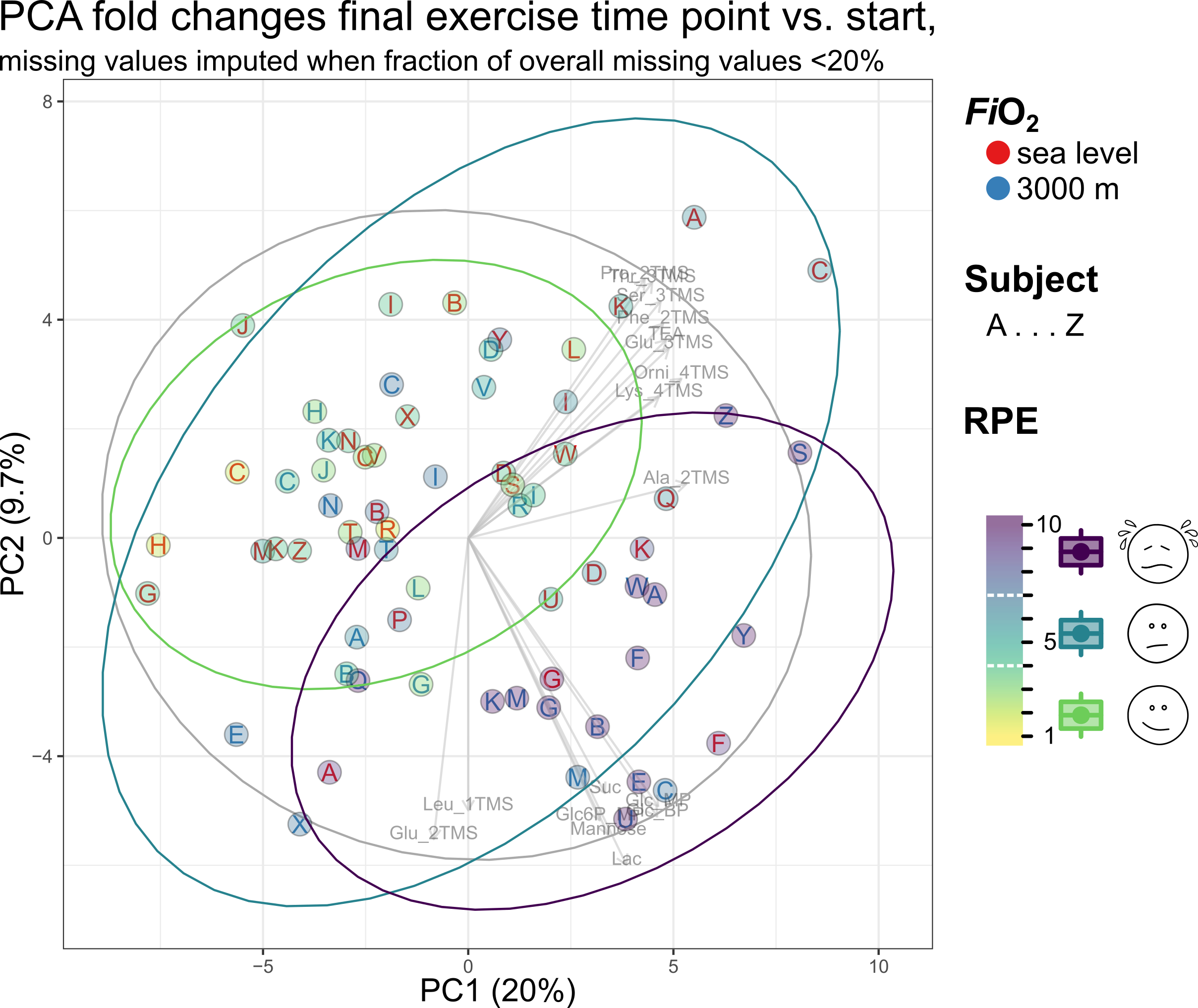


Figure S8 Related to figure 2G-I, PCA of fold change of metabolite intensities from baseline to final exercise time point including members of all RPE subgroups. Areas are colored by RPE, letters depicting individuals are colored according to oxygenation state. Extreme outliers (not shown) according to the T-ellipse (gray) were removed, and PCA was re-run.

##
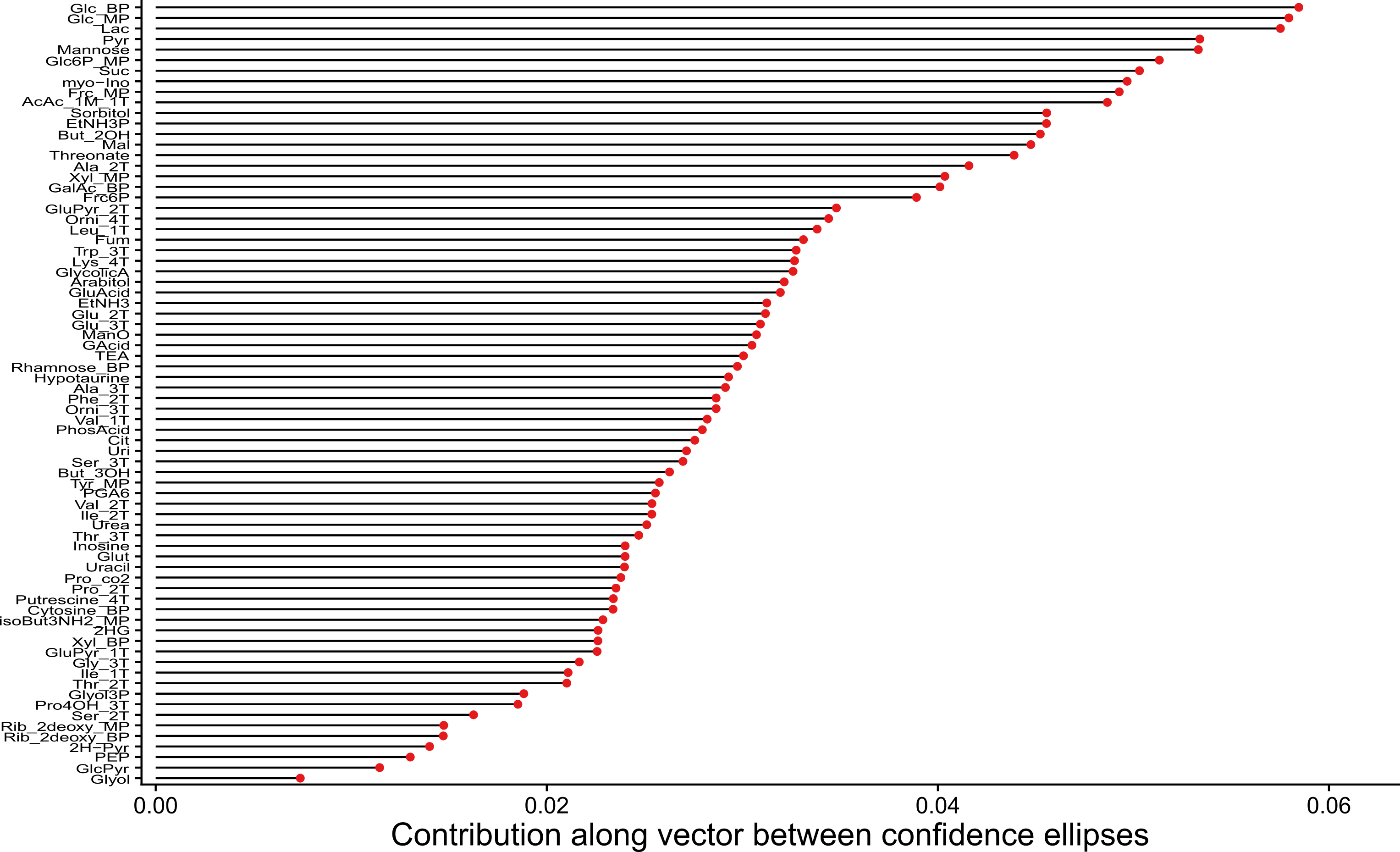
Figure S9 Dogleg plot of Metabolites from PC1, Figure S8

Figure S9 Related to figure 2G-I, Loadings for metabolites in principal component 1 (PC1), performed for all RPE-subgroups. Glucose has a high influence as well as alanine, mannose and lactate and pyruvate, indicating a highly active Cahill-cycle.

## Figure S10 Distribution between ellipses centers when including all RPE groups

**
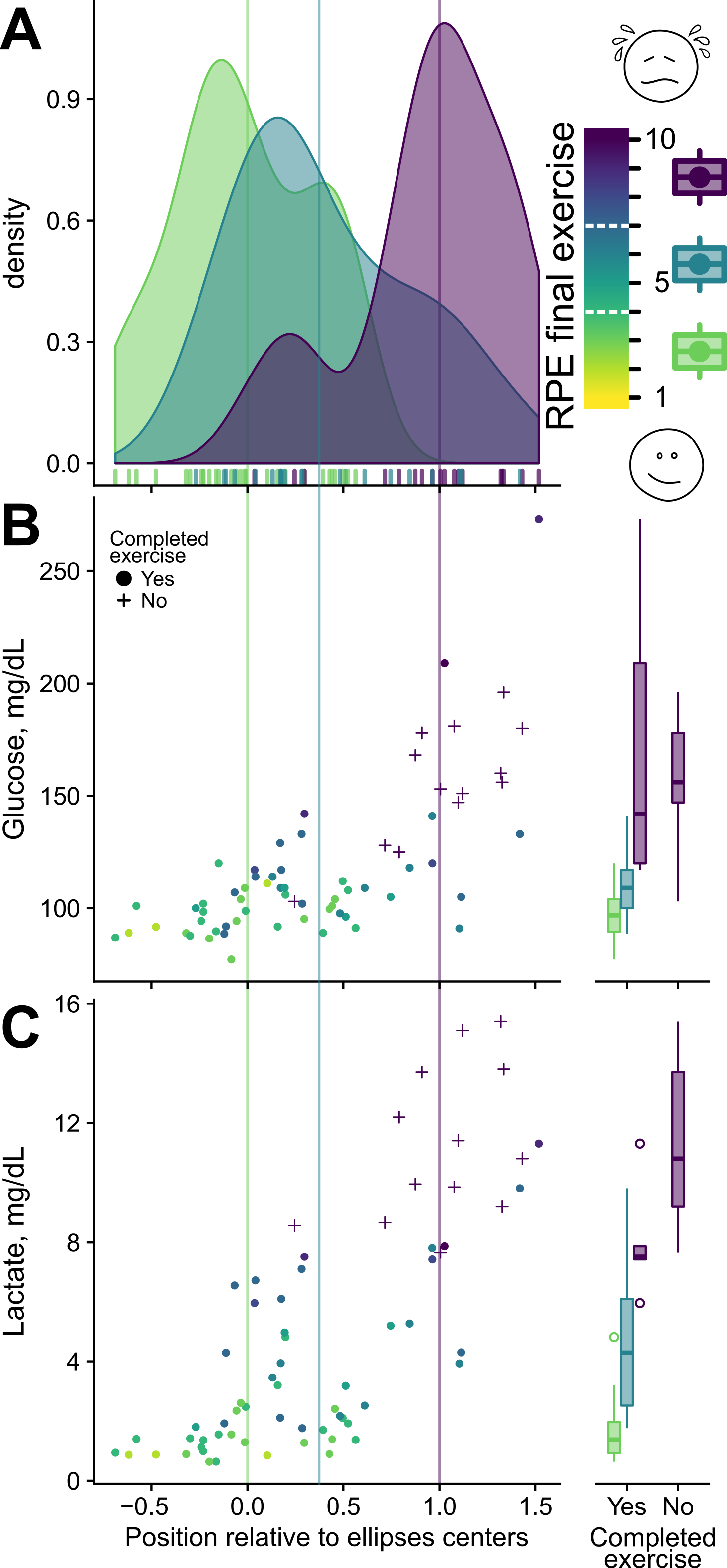
**

Figure S10 Related to figure 2G-I, Distribution of samples along ellipses centers from PCA of samples when all RPE groups are included with the most important separators (glucose (A) and lactate (B) shown exemplary). Some samples do not separate clearly from the other RPE groups (dotted line). However, if those separated by PCA are filtered and PCA is re-run on the reduced data set, we find a similar, less pronounce phenotype (see S.PCA.subgroup).

## Figure S11 Distribution along PC1 when including all RPE groups and filtering those from highest RPE group already separated in Figure S10A

**
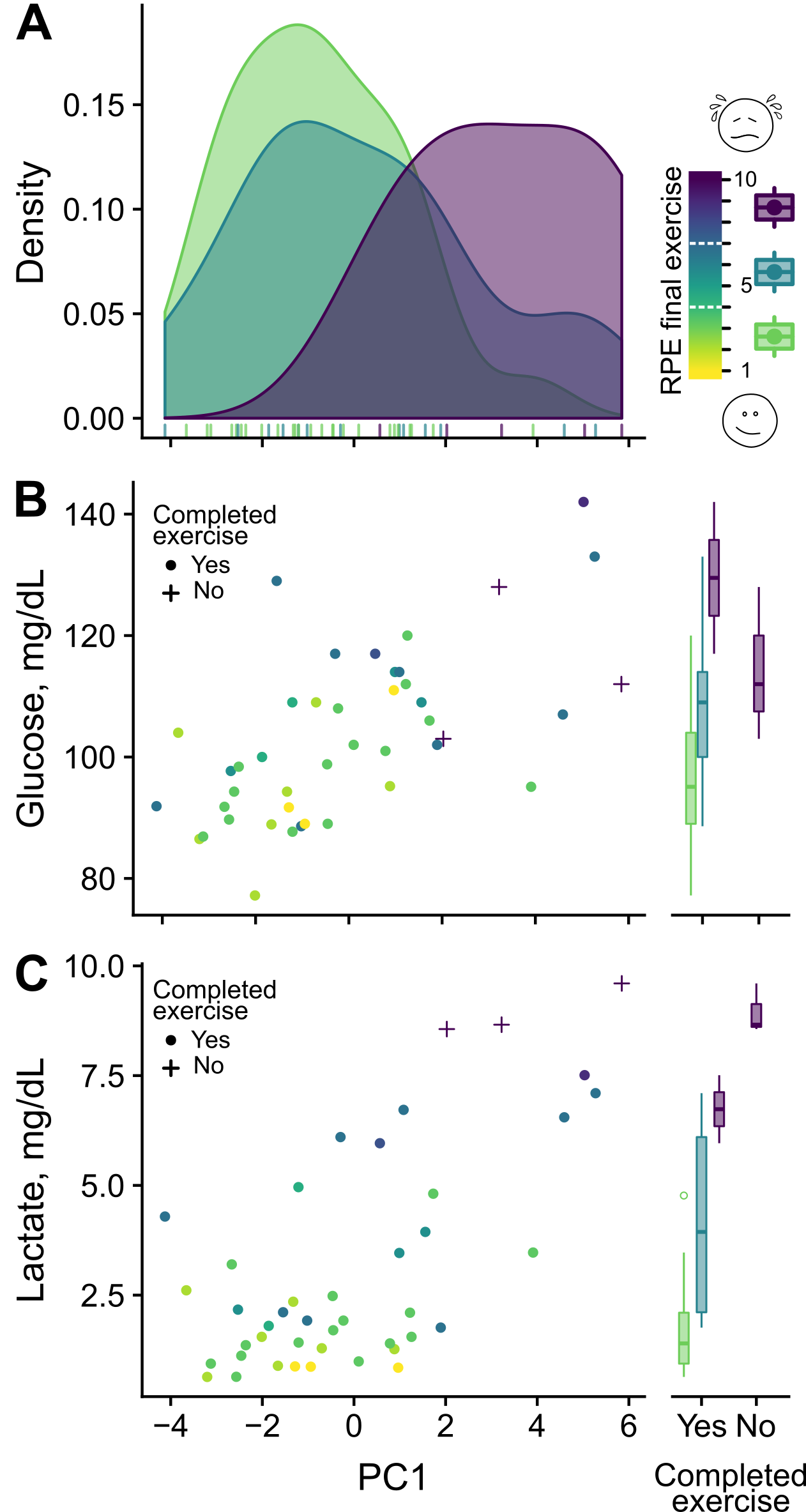
**

Figure S11 Related to figure 2G-I, PCA of samples when initially separated group of subjects arriving at high exertion is filtered from the data set (dotted line). The samples that grouped with samples from lower RPE groups now separate from samples of less exerted individuals, but show a less pronounced phenotype in glucose and lactate.

## Figure S12 Distribution along ellipses centers according to *Fi*O_2_ levels including all RPE groups


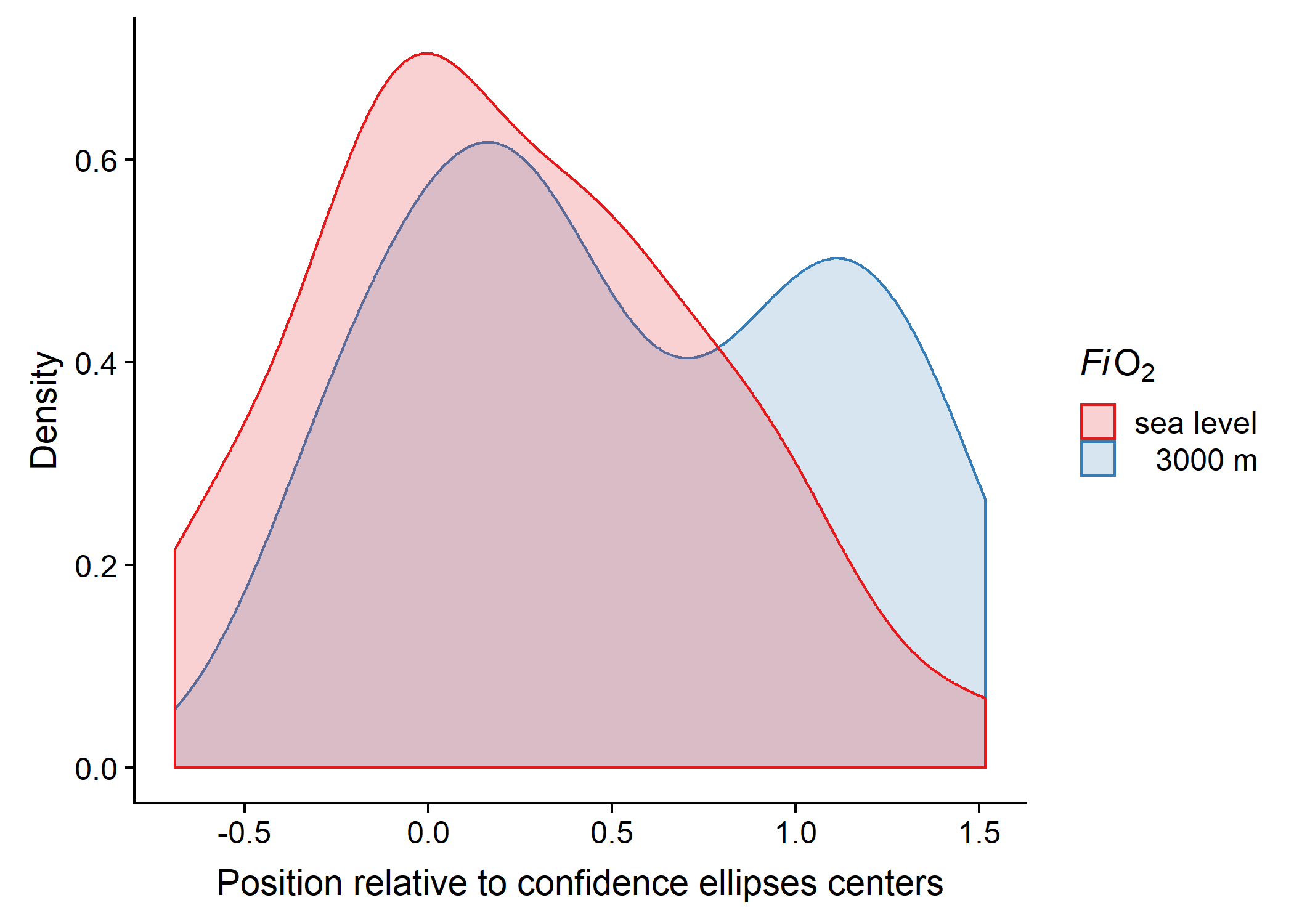


Figure S12 Related to figure 2G-I, Plot of distribution of samples along vector connecting confidence ellipses centers (see Figure S8, S10) obtained under different FiO_2_ levels when samples from all RPE-groups were included. There is a slight separation, but this likely appears, since exercise is more strenuous under hypoxic conditions, so a slight bias is to be expected.

## Figure S13 Lineplots of metabolite fold changes showing all RPE groups


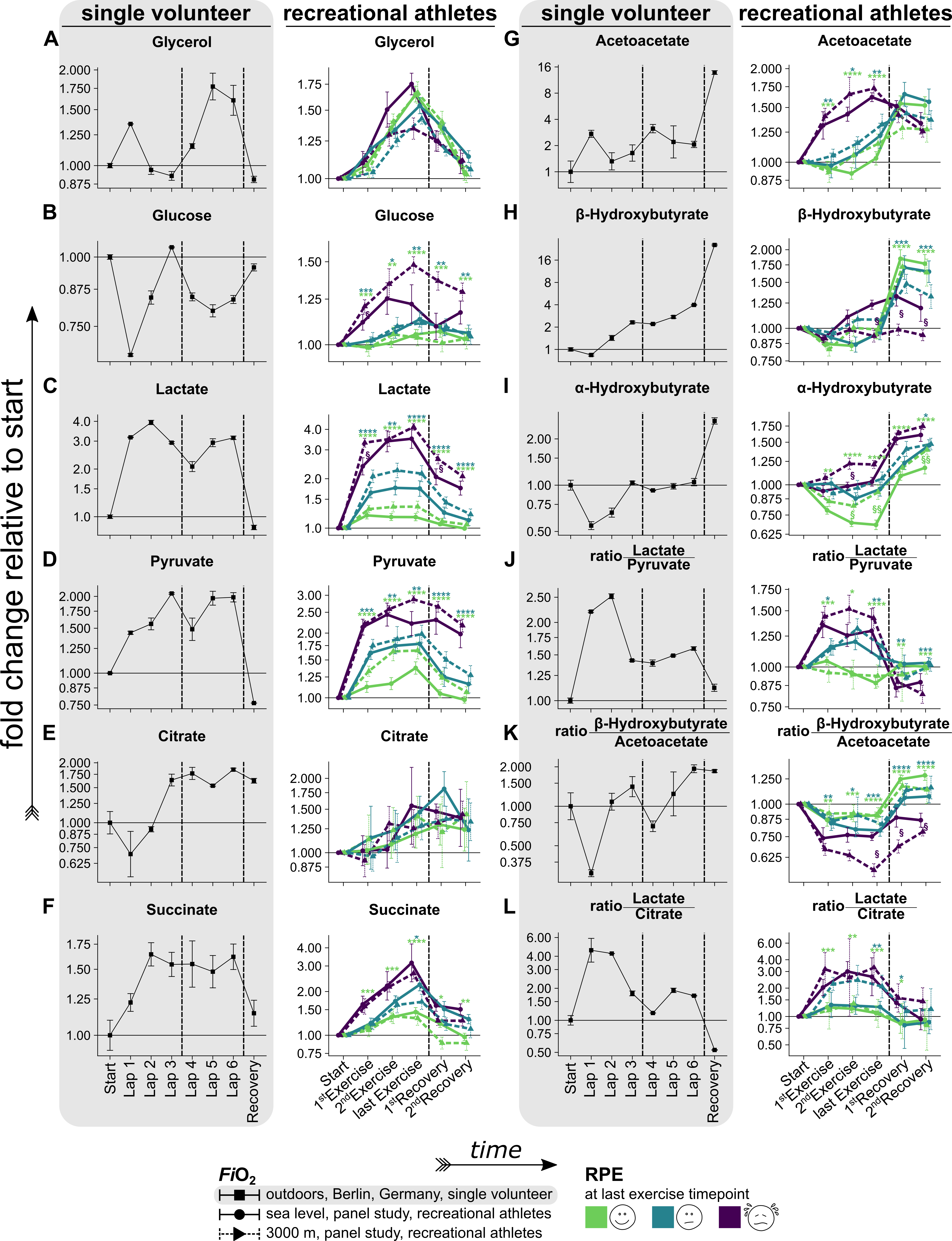


Figure S13 Related to figure 3, showing time profiles of central carbon metabolites (A-F), ketone bodies (G-I) and metabolite ratios (J-L) in a single volunteer (black, left, error bars: minimum and maximum values per time point) or grouped according to FiO_2_ (solid: sea level, dotted: 3000 m) and RPE at last exercise time point (coloured, right, error bars: SEM). Dashed lines encompass comparable time intervals in different setups. In panels on the left only one recovery time point was measured, while in the right panels “hitting the wall” was never overcome, but exercise was stopped after 30 min. Note different scales on ordinates between experiments and logarithmic intervals on the ordinate axes. p-values from Wilcoxon-rank-sum test (Benjamini-Hochberg corrected) * p<0.05; ** p<0.01; *** p<0.001; *** p<0.0001 of all values within one group of RPE, for clarity only significance vs lowest RPE group is shown, but all comparisons were accounted for p-value correction, § p<0.05; §§ p<0.01 between hypoxia and normoxia of lowest RPE group. Explanations in the main text. Note that the group with the „median“ RPE-level tends to fall always in between the two more extreme groups.

## Figure S14 TCA intermediates in elite athlete


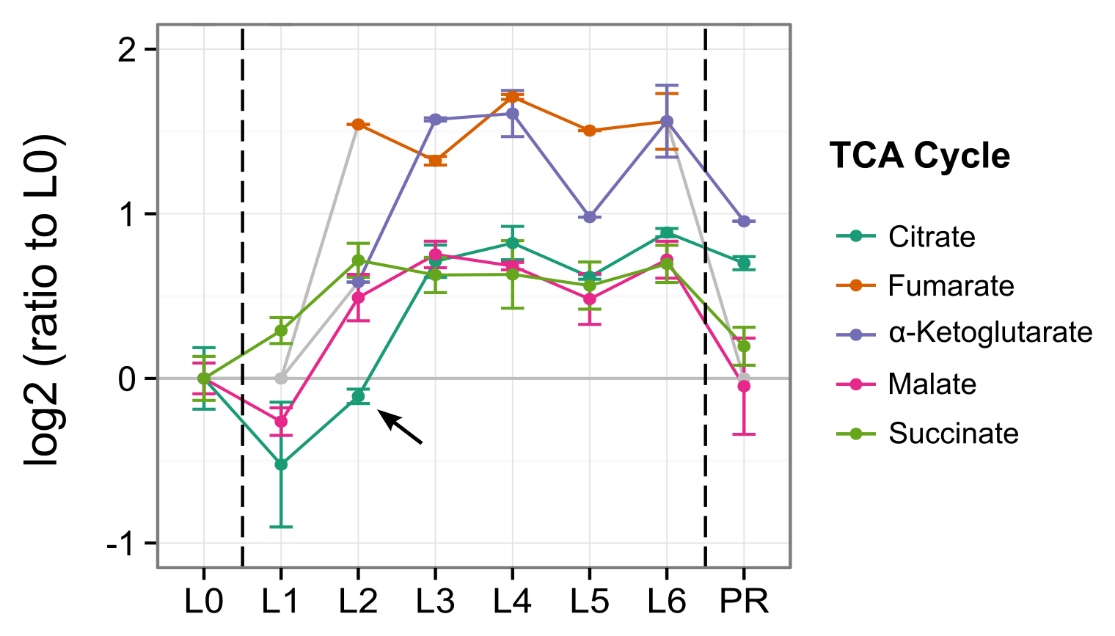


Figure S14 Related to figures 1&3, All identified TCA cycle intermediates rose to their highest levels by L2 except for citrate (arrow). Citrate reached high levels in L3 after oxygen release of erythrocytes.

Gray points and lines indicate the metabolite to have been below detection in the sampling time point.

Metabolite levels are represented as log2 values of the ratio of each sampling time point to the L0 sample (except for fumarate and α-ketoglutarate, which were below the detection in L0. Their levels have been transposed so that they can be visualized with the others). Error bars indicate minimum and maximum values for each time point.

## Figure S15 Free Fatty Acids and glycerol in elite athlete


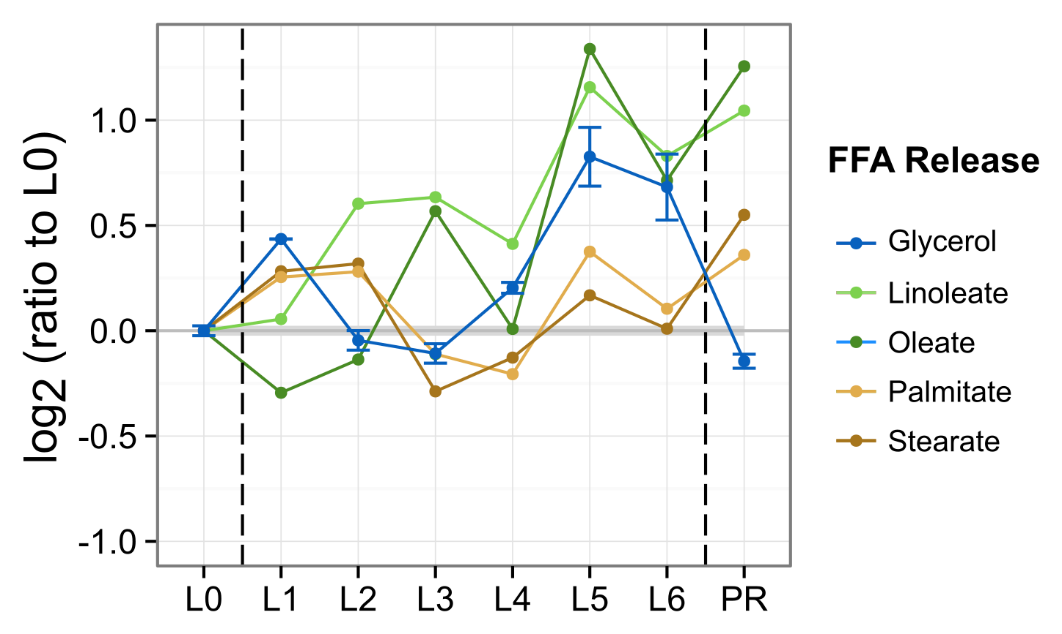


Figure S15 Related to figure 3, Glycerol levels and the four most abundant FFAs indicated two phases of release, one occurring immediately after exercise initiation and the second after the “high” in L4. Note that oleate and linoleate levels, the two most abundant unsaturated FFAs, rose delayed relative to palmitate and stearate in the first release phase, and rose much more strongly in the second phase.

Metabolite levels are represented as log2 values of the ratio of each sampling time point to the L0 sample (except for fumarate and α-ketoglutarate, which were below the detection in L0. Their levels have been transposed so that they can be visualized with the others). Error bars indicate minimum and maximum values for each time point.

## Figure S16 TCA intermediates in subject panel


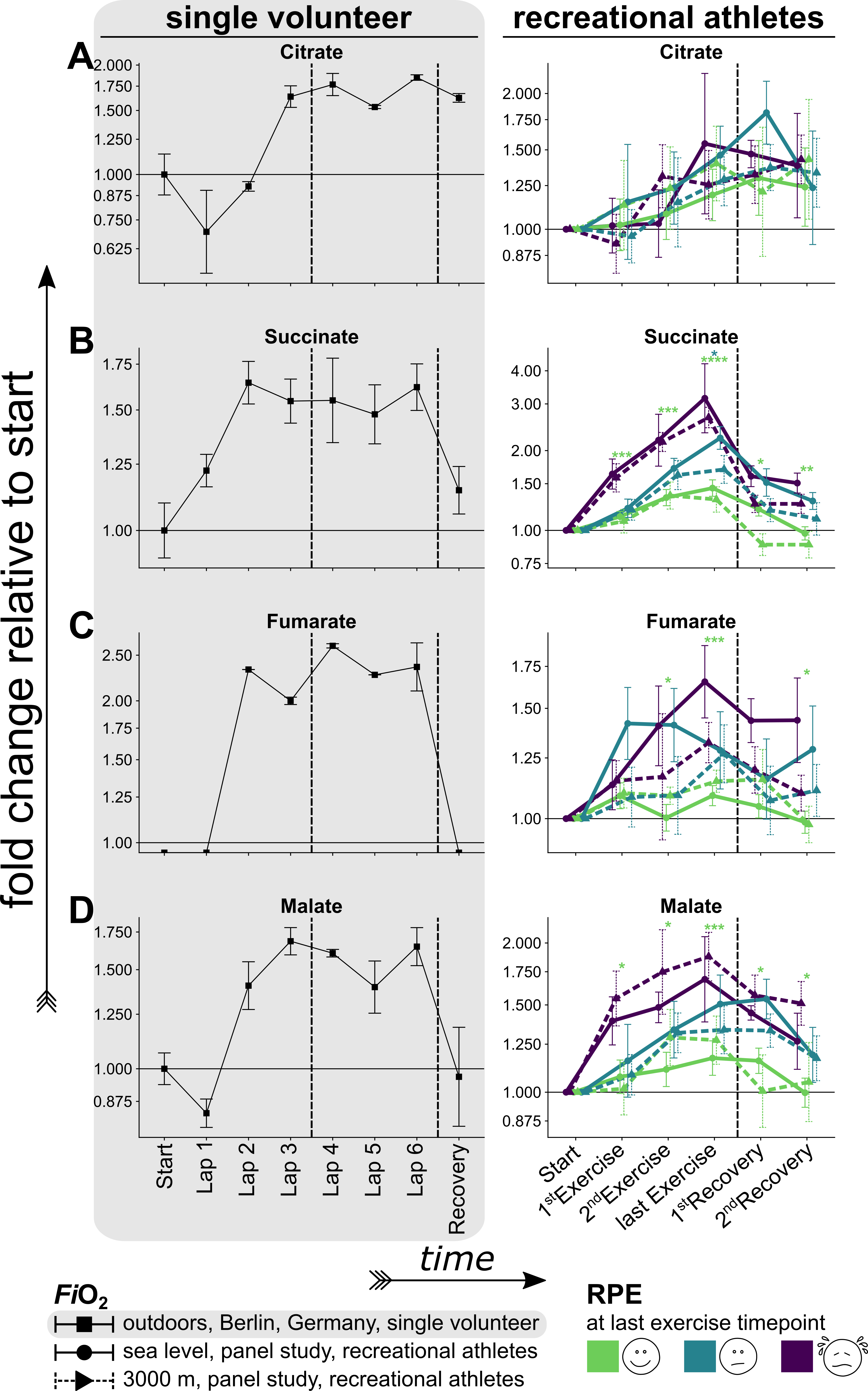


Figure S 16 Related to figure 3, comparison of time profiles of TCA intermediates (A-D) in a single volunteer (black, left, error bars: minimum and maximum values per time point) or grouped according to FiO_2_ (solid: sea level, dotted: 3000 m) and RPE at last exercise time point (colored, right, error bars: SEM), legend see also Figure 3.

## Figure S17 Correlation plot Glucose vs. αOHB


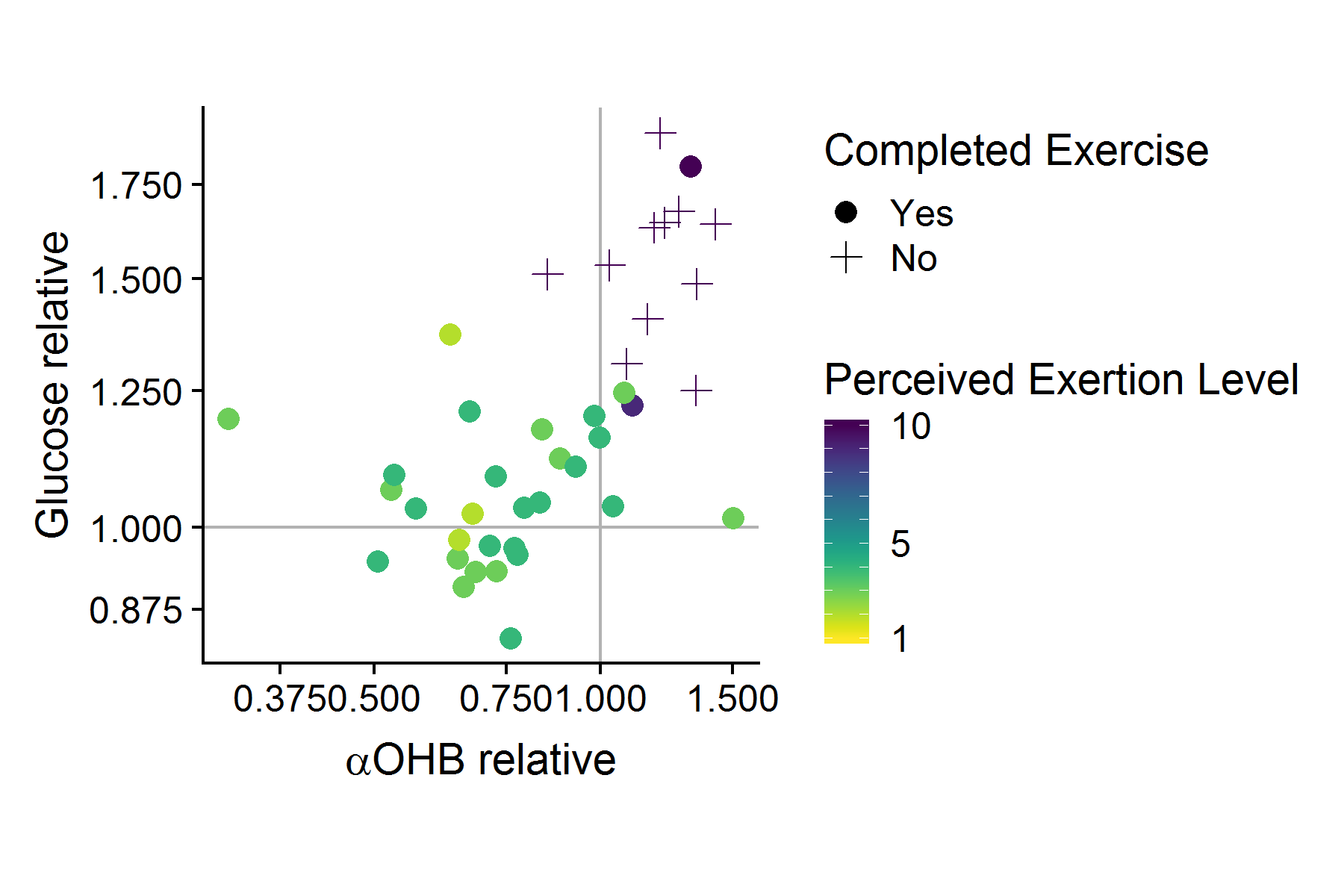


Figure S17 Related to figure 2H, correlation of α-hydroxybutyrate and glucose at last exercise time point; Axes show fold changes relative to baseline, note the log scale.

##
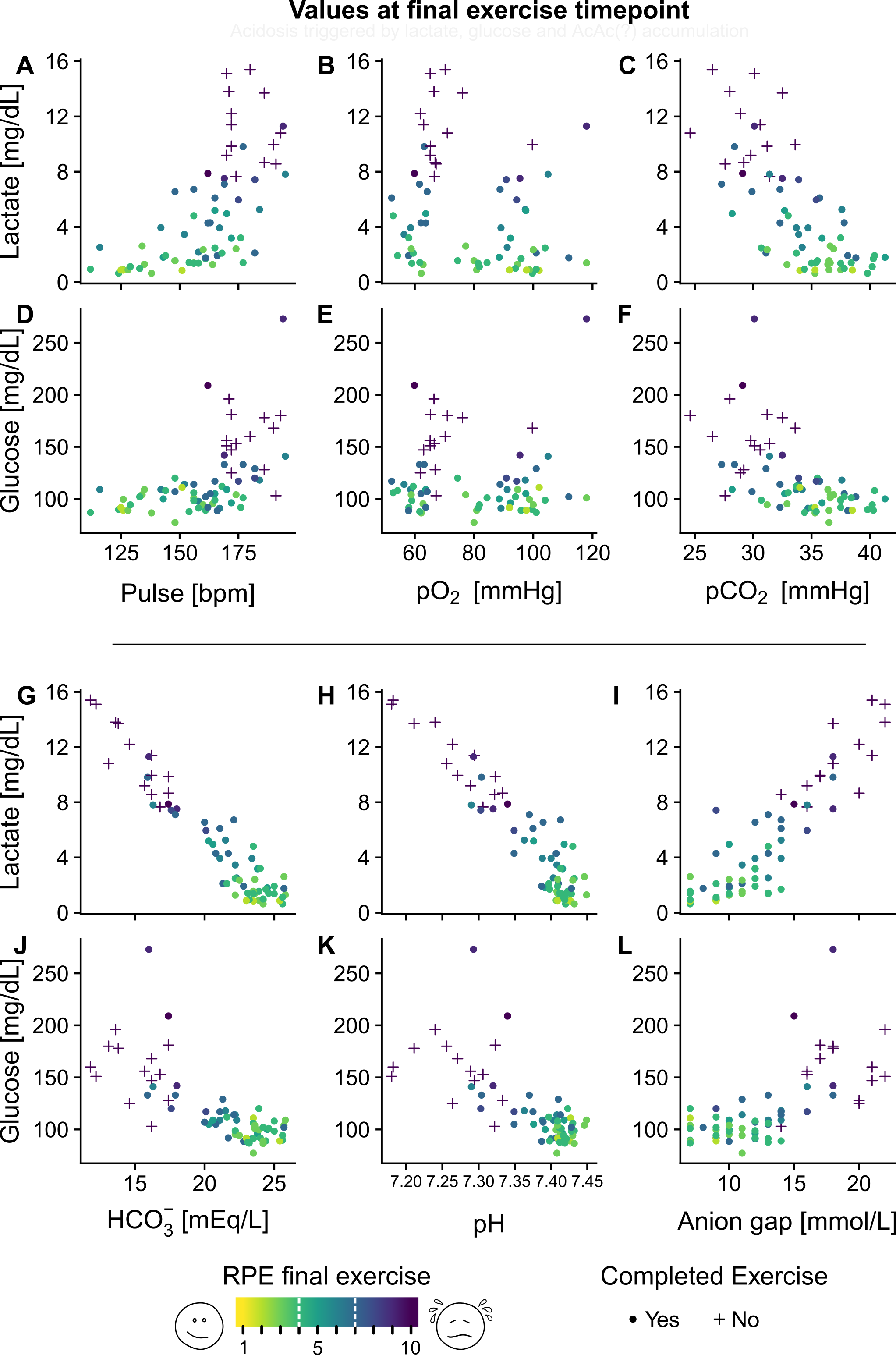
Figure S18 Correlations of Lactate and Glucose with BGA measures

Figure S18 Related to figure 2H–I, correlation of several clinical measurements with lactate and glucose amounts at final exercise time point, all RPE groups included. They show correlation and the pH-related measures reflect likely metabolic acidosis (low pH, pCO2, HCO3 and high anion gap, which all coincide with high acetoacetate (Figure 3G), glucose (Figures 2H, 3B) and lactate (Figures 2I, 3C). The glucose relationship is less obvious. Also in diabetics, often diagnosed with metabolic acidosis, usually “total” ketone bodies are determined. Note, that we see in these data, that acetoacetate is high (Figure 3G), while β-hydroxybutyrate is low in hypoxia (Figure 3H), but higher in normoxia in the high RPE-group, while the α-hydroxybutyrate, which is associated with development of diabetes, is increased.

## Figure S19 Profiles of Alanine levels


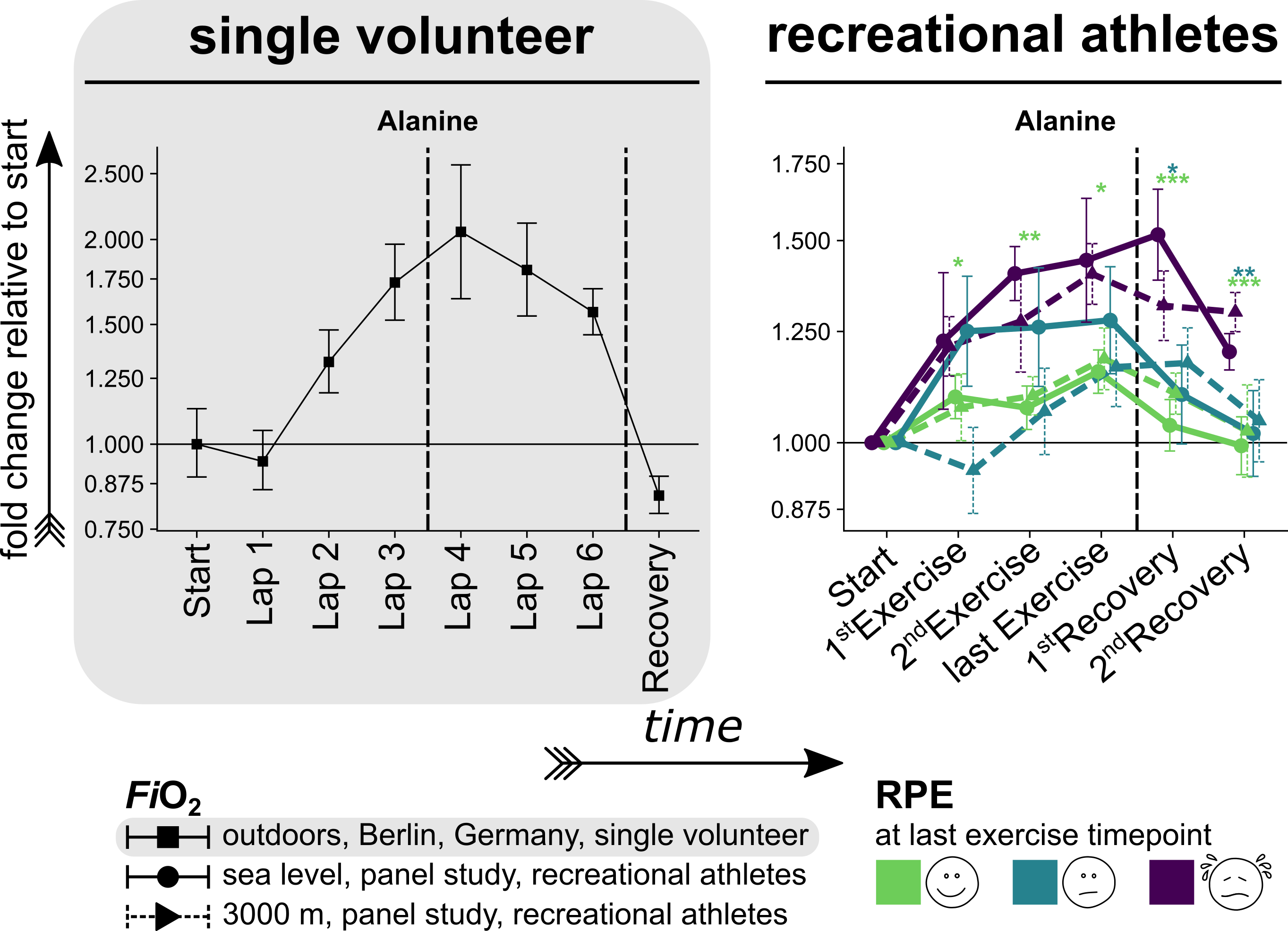


Figure S19 Related to figure 3, time profile of alanine in single volunteer (left) and subject panel (right). Legend see Figures 3 or S13.

## Figure S20 Profiles of Glucose-6-phosphate levels


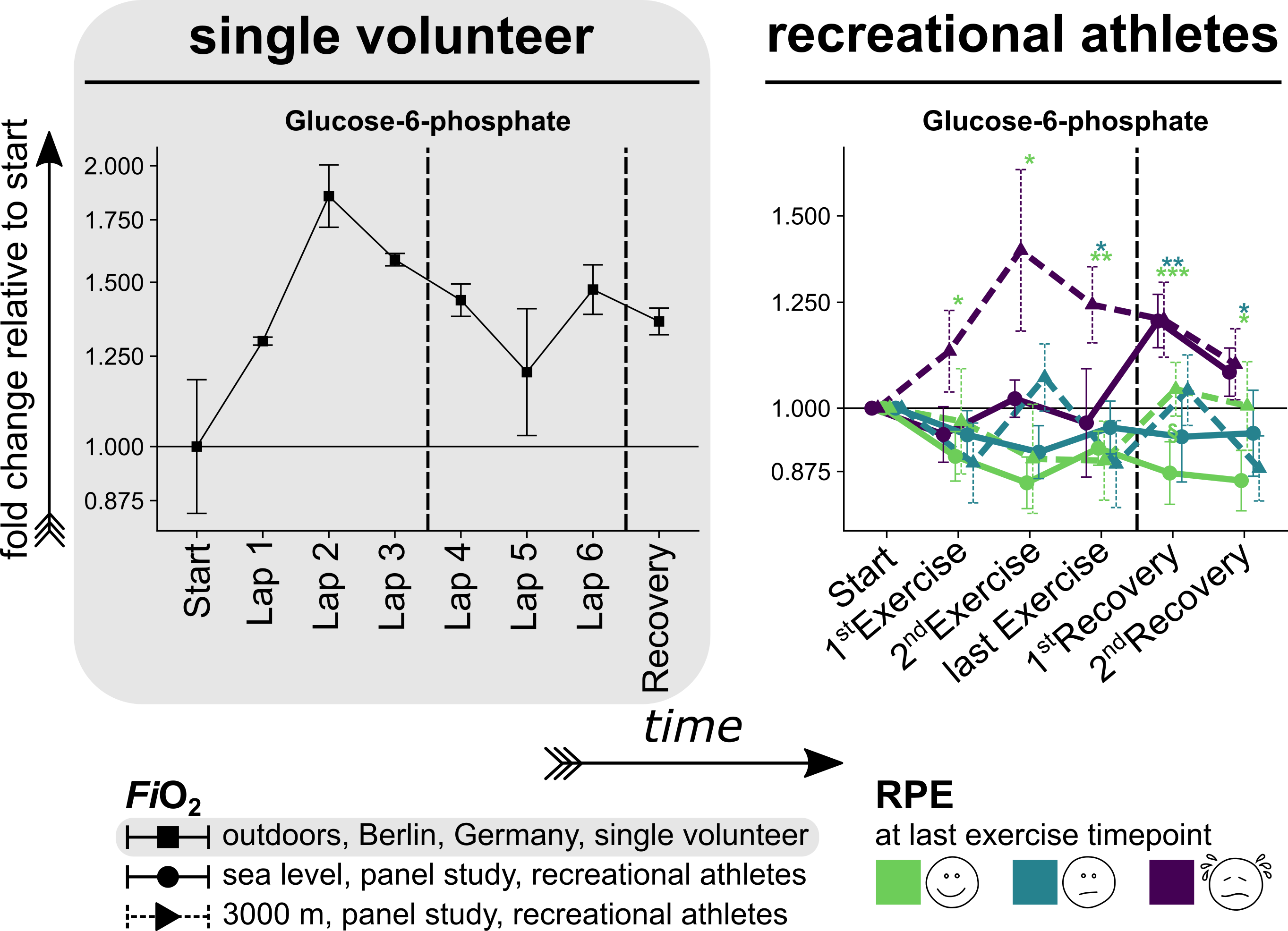


Figure S20 Related to figure 3, time profile of glucose-6-phosphate in single volunteer (left) and subject panel (right). Legend see Figures 3 or S13. Note that glucose-6-phosphate rises similarly in the highly challenged group and the single volunteer, pointing to highly active glycolysis. The single volunteer seems however better equipped or able to deal with the resulting accumulation of lactate (figure 3C)

## Figure S21 Correlation of glucose and lactate quantification by different techniques


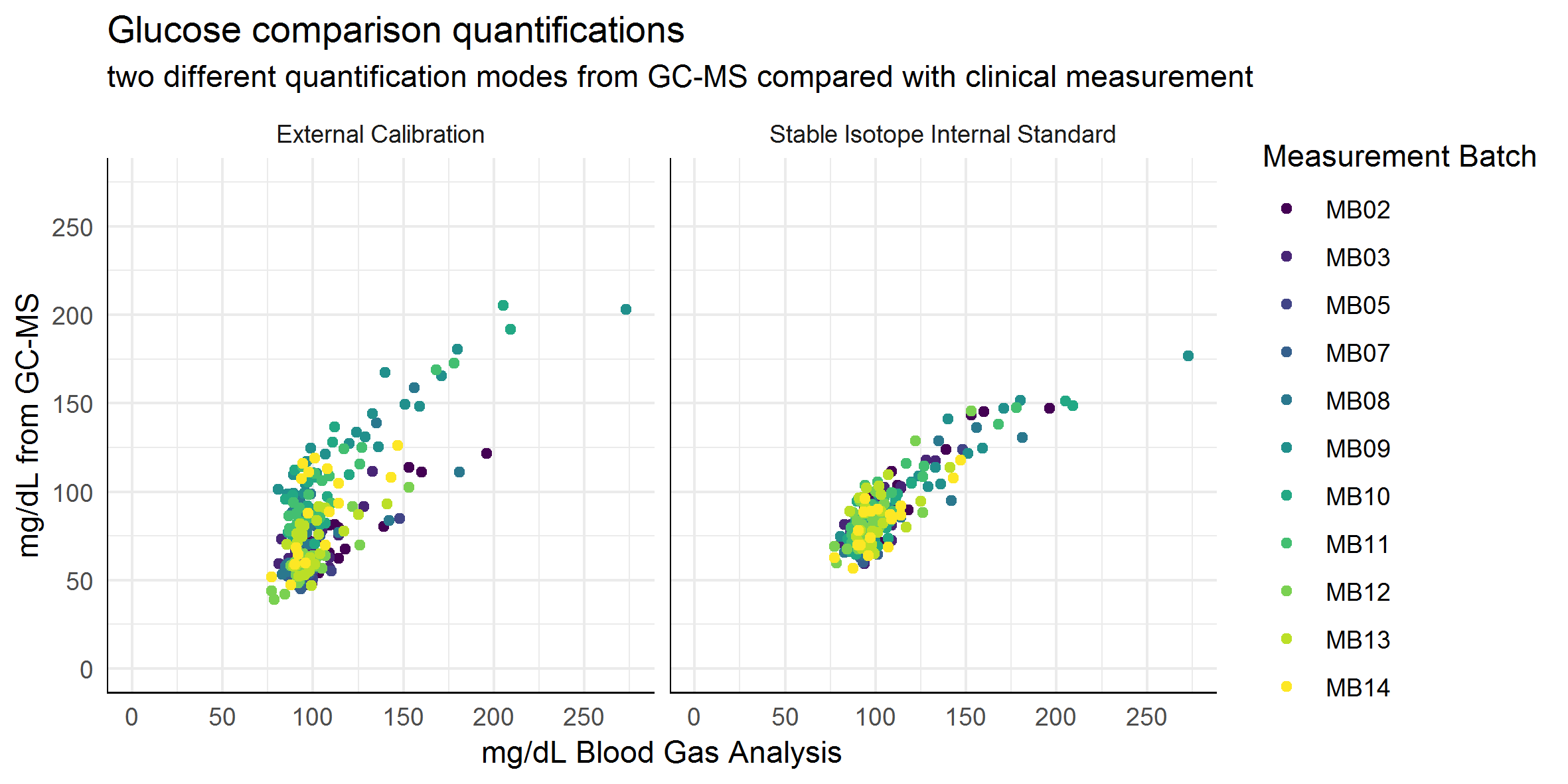


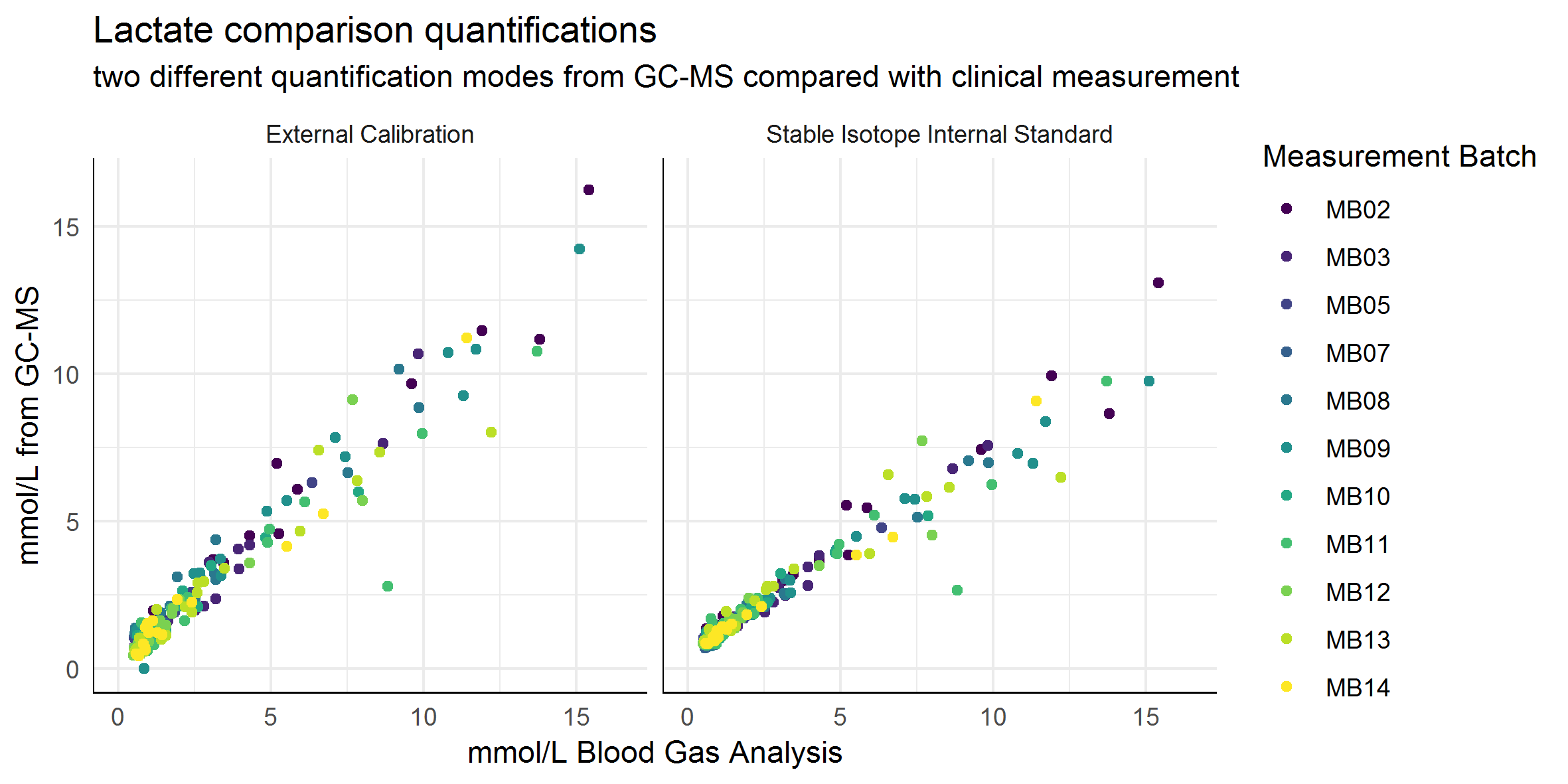


Figure S21 Related to figures 2H&I, 3B&C, Correlation of Blood gas analysis sample measured by commercial analyzers from a clinical setting and our GC–MS based approach. A trend or bias according to specific GC–MS measurement batches is not found. As expected quantification with internal stabile isotope standard works best for both metabolites. While quantification by external calibration correlates very well with standard clinical measurement for lactate, for glucose we see likely an underestimation for lower values in external calibration. In this case the clinical measurements are more likely “real” values, because they fit better to established physiological values. Lactate is measured systematically slightly lower with internal standard quantification, most likely because it isn’t corrected for background from naturally occurring heavy isotopes.

## Figure S22 Missing values according to *Fi*O_2_ and sex


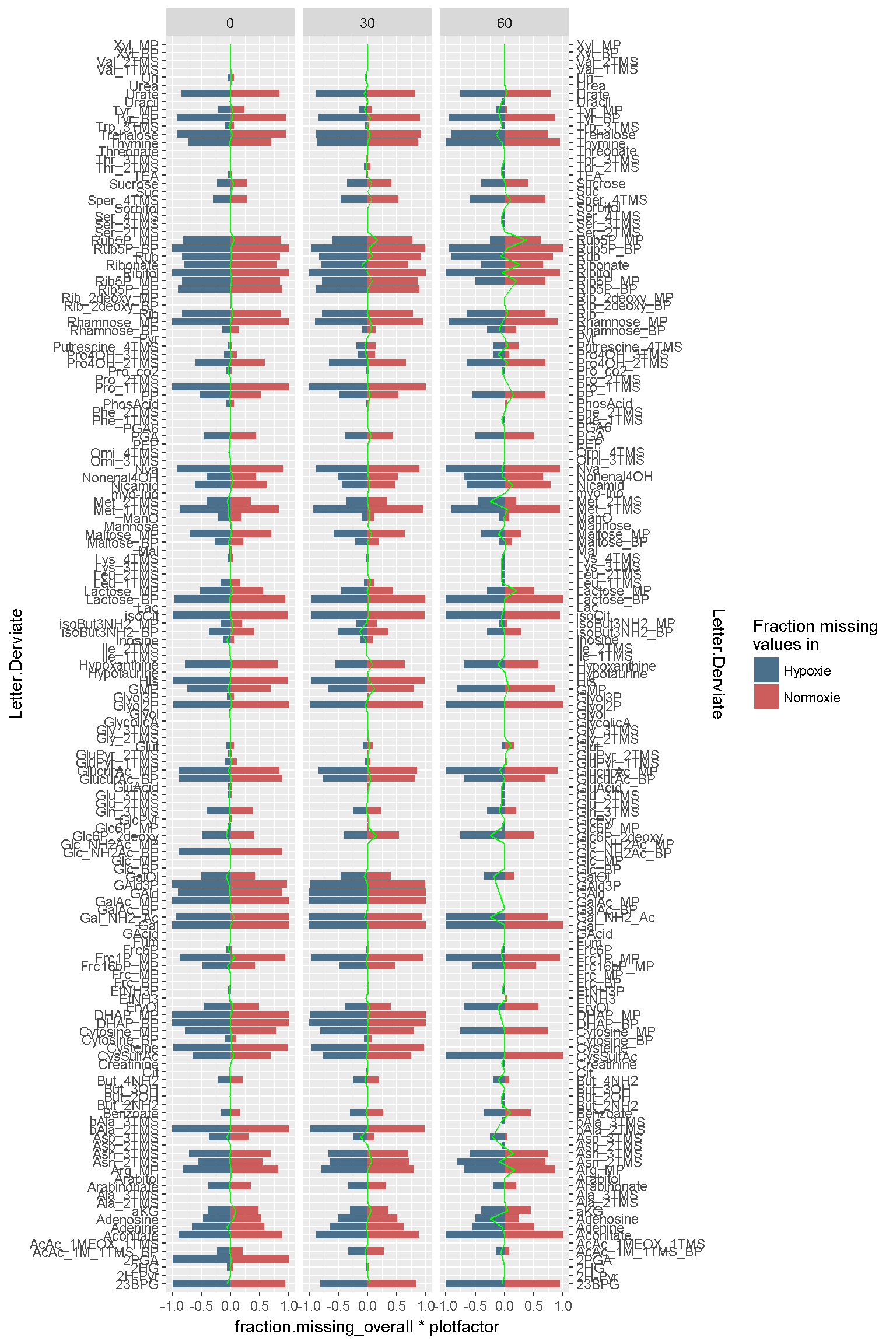


**A**


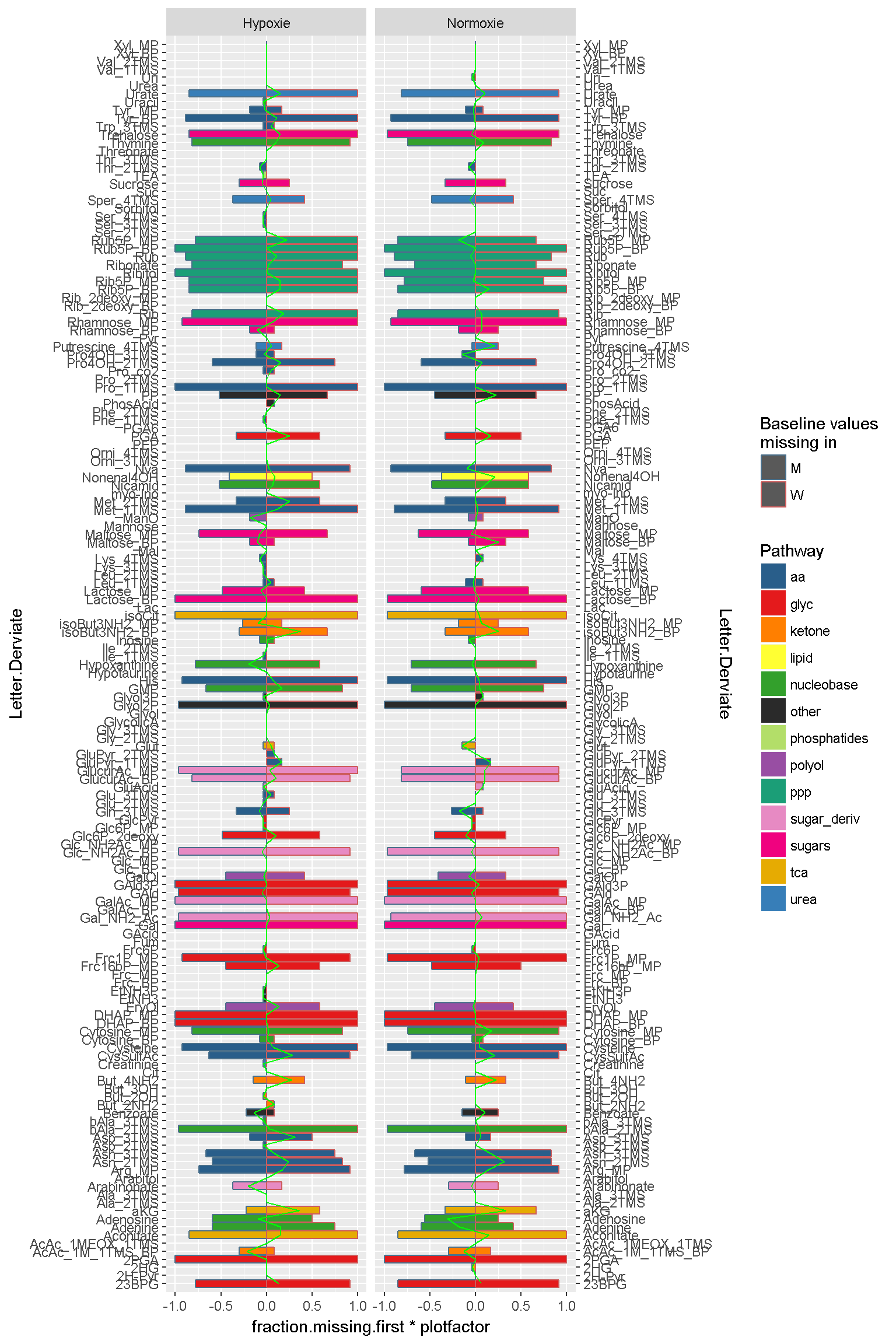


**B**

Figure S22 Related to figure 2G, distribution of missing values according to running speed or sex and FiO_2_. Except for clear oxygenation markers accordingly with FiO_2_ and effort level (ribose-5-phosphate and ribulose-5-phosphate) and iso-aminobutyrate in females, no compound was significantly missing more in one condition.

## Figure S23 Oxygen markers


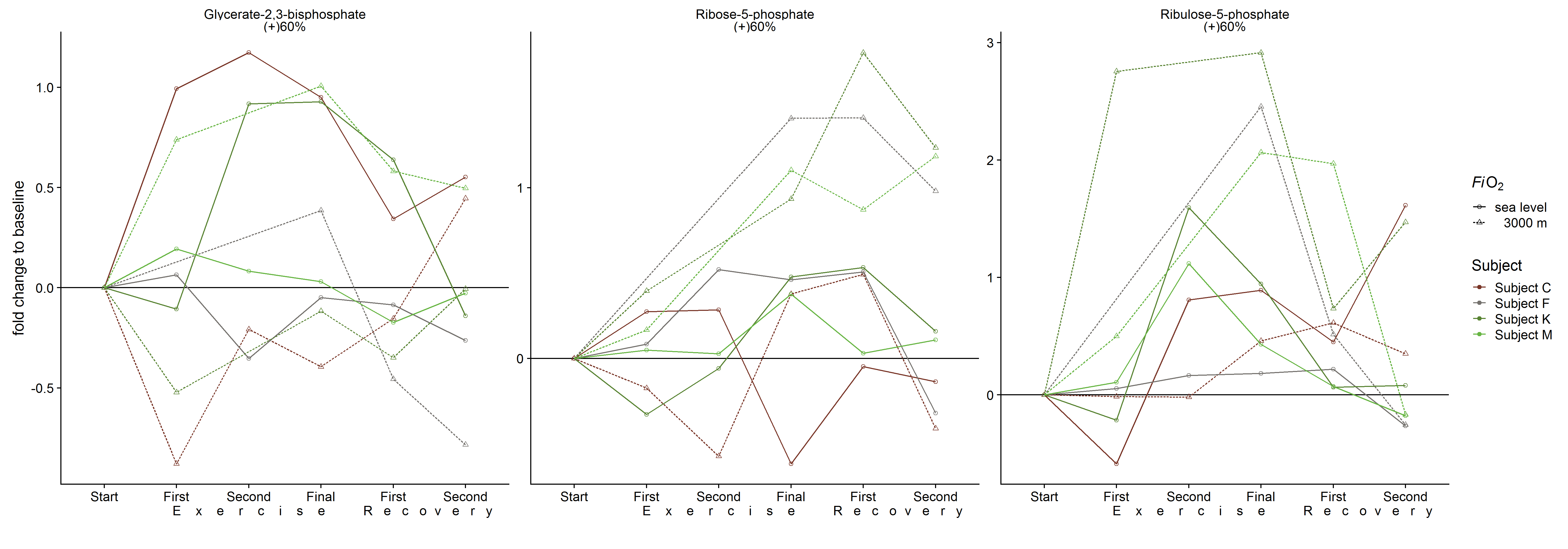


Figure S23 Related to figure 3, Data from 4 Subjects performing at 1.6 fold the calculated rate. Solid lines: Sea level, dotted lines: 3000 m. In some exercise bouts and subjects Ribose 5P and Ribulose 5P are formed; more under hypoxic than normoxic conditions, Ribose 5P stays elevated under resting hypoxia. 2,3‑BPG is formed only in certain exercise bouts. Y-axis values are log_2_-foldchanges (0= baseline, 1 means a twofold change (2^1^), 3 means 8 fold (2^3^)

One subject that shows relatively little formation of the PPP-intermediates shows a strong increase of 2,3‑BPG under normoxia, but not hypoxia.

Overall data is not conclusive in relation to our single human volunteer, it should be noted, that the secondary experiment was not designed towards “overcoming the wall”. Overall intensities in the samples were relatively low, so quantitation is not entirely reliable.. Maybe 2,3‑BPG also “senses” external O_2_, or there is a somewhat compensatory effect with PPP?

## Table S6 Stable isotopes added as internal standard during extraction

Table S6 List of stable isotopes added to the samples during extraction, and mass pairs deemed to include label. We only used the internal standards from glucose and lactate to compare our method to a clinical routine analysis.

| substance name | Light ion | Heavy ion | µg/Sample |
| --- | --- | --- | --- |
| Glucose ^13^C_6_ | 319  217 | 323  220 | 31.360 |
| Sodium L-Lactate ^13^C_3_ | 117  190 | 119  193 | 17.920 |
